# Dynamic chromatin accessibility reveals BrKAN2 as a key regulator of Chinese cabbage leaf heading

**DOI:** 10.1186/s43897-026-00239-6

**Published:** 2026-07-08

**Authors:** Huiling Guo, Fengming Li, Lupeng Zhang, Yuanyuan Zhang, Xu Cai, Haixu Chen, Xiaoxiao Zheng, Jian Wu, Xiaowu Wang, Jianli Liang

**Affiliations:** State Key Laboratory of Vegetable Biobreeding, Institute of Vegetables and Flowers, Chinese Academy of Agricultural Sciences, Beijing, 100081 China

**Keywords:** Chinese cabbage, Leaf heading, ATAC-seq, BrKAN2, DAP-seq

## Abstract

Chinese cabbage forms a leafy head as its main edible organ, a process involving extensive morphological and transcriptional changes. Here, we employed ATAC-seq to profile a time-series chromatin accessibility landscape across key developmental stages of leaf heading. This analysis revealed highly dynamic, stage-specific chromatin accessible regions accompanied by distinct transcription factors activities. Genes associated with adaxial-abaxial polarity and hormone signaling displayed dynamic accessibility patterns, suggesting their critical roles in head formation. BrKAN2 emerged as a key candidate regulator. Mutant analysis in Chinese cabbage and overexpression in *Arabidopsis* significantly altered leaf morphology and dorsoventral polarity. Population genetic analysis further indicated strong selection on *BrKAN2* in heading *Brassica rapa*. Integration of DAP-seq and ATAC-seq identified BrKAN2.1 target genes enriched in organ development and hormone-related pathways, which were validated by VIGS and EMSA. Auxin responsiveness assays underscored the importance of auxin signaling in heading. Collectively, these findings uncover a dynamic chromatin landscape underlying leaf heading and establish BrKAN2 as a central regulatory factor, offering new insights for genetic improvement of Chinese cabbage.

**Supplementary Information:**

The online version contains supplementary material available at 10.1186/s43897-026-00239-6.

## Core

Among genes associated with adaxial-abaxial polarity and hormonal signaling, *BrKAN2* genes were identified as critical regulators. Functional analysis in both Chinese cabbage and *Arabidopsis* confirmed their roles in leaf morphology and dorsoventral polarity. Integrative DAP-seq and ATAC-seq analyses further identified BrKAN2.1 target genes enriched in auxin related pathways, suggesting its role in coordinating polarity and auxin signaling during head formation.

## Genes & accession numbers

All sequencing data generated in this study have been deposited in the NGDC Sequence Read Archive (https://ngdc.cncb.ac.cn/) under accession number PRJCA033732.

## Introduction

Chinese cabbage (*Brassica rapa* ssp*. pekinensis*), a major leafy vegetable in East Asia, is valued for its unique leafy head, which determines both yield and market quality. The development of the leafy head is a complex, highly coordinated process involving distinct morphological and transcriptional transitions. This process can be divided into three major stages: seedling stage, rosette stage and heading stage. At seedling stage, the plant initiates photosynthesis, typically producing 5-8 leaves arranged in 2-3 whorls around the shoot apical meristem (SAM). Notably, vertical growth remains subdued during this phase. In the subsequent rosette stage, the existing leaves expand fully. Outer leaves gracefully assume a horizontal orientation, while fresh foliage emerges around the SAM. Simultaneously, the inner leaves commence their upward growth. Finally, in the heading stage, the leaves persist in their upward growth, gradually curving inward. This process culminates as the leaves tightly coverage to form the distinctive leafy head (Sun et al. 2019).

Leaf heading is driven by extensive changes in gene expression and cellular architecture. A series of transcriptomic studies on Chinese cabbage leaves at various stages of leafy head development has unveiled the meticulous control exerted by an intricate transcriptional network, which integrate internal signals with external environmental cues. Several key factors implicated in this regulatory network have been identified, including hormones such as auxin, cytokinin, abscisic acid, gibberellin, and brassinosteroids, as well as environmental stimuli such as temperature, light and carbohydrate levels (Wang et al. 2012; Gu et al. 2017; Li et al. 2019; Gao et al. 2020; Guo et al. 2022). Recent work has identified a critical transition stage that initiates heading in Chinese cabbage. This transition phase is characterized by a pronounced upregulation of genes related to phytohormones, MAPK pathways, pivotal transcription factors (TFs), and genes involved in leaf adaxial-abaxial pathway, as well as those regulating polar auxin transport, cell differentiation and division (Zhang et al. 2022a).

In the context of the leafy head domestication trait, several genes associated with adaxial-abaxial polarity pathway, including the *ARF3*, *ARF4*, *KAN2*, and *ATHB15* genes, were found to be under selection in leaf heading accessions, indicating their pivotal role in leaf heading (Liang et al. 2016; Cheng et al. 2016). Genetic analysis have highlighted the impact of the adaxial-abaxial polarity pathway gene *BcpLH* on the inward curvature of Chinese cabbage leaves (Ren et al. 2020). Additionally, key TFs, such as BrTCP4 and BrSPL9, have been identified as key players in leafy head formation, influencing head shape and heading time, respectively (Mao et al. 2014; Wang et al. 2014). Investigation into Chinese cabbage mutants lacking head formation unveiled the involvement of the gibberellin biosynthesis related gene *BrKS1* in the process of leafy head formation (Gao et al. 2020). Recent study characterized an inward curling mutant, elucidating the positive regulatory role of *BrOPS* in BR signaling by antagonizing *BrBIN2* to promote leaf curvature in Chinese cabbage (Zhang et al. 2022a, b). The role of auxin biosynthesis genes (AUXs) in leafy head formation has also been explored, suggesting their potential involvement in regulating auxin concentration (He et al. 2000). Moreover, the *BrAN *genes were revealed to control leafy head formation by regulating leaf width or pavement cell shape in Chinese cabbage (Xin et al. 2022).

The mechanism governing leaf adaxial-abaxial polarity specification and the development of a flat lamina has been extensively investigated. This process involves a complex interplay of TFs and small RNAs that intricately regulate the leaf adaxial-abaxial polarity. Adaxial polarity is primarily determined by the class III homeodomain-leucine zipper (HD-ZIPIII) family of TFs, including *REV*, *PHB*, *PHV*, and *ATHB8 *(Emery et al. 2003), as well as MYB and LOB domain TFs such as *AS1* and *AS2 *(Iwakawa et al. 2007), and trans-acting short interfering RNAs (TAS3-derived *tasiR-ARF*). In contrast, the abaxial domain is characterized by the KANADI family genes (*KAN1-3*) (Kerstetter et al. 2001; Eshed et al. 2001, 2004), auxin response factors (*ARF*) *ETTIN* (*ETT*/*ARF3*) and *ARF4 *(Pekker et al. 2005), *YABBY* family genes (*YAB1* to *YAB3*) and *miR165/166* small RNAs (Kidner and Martienssen 2004).

The *KAN* genes (*KAN1* to *KAN4*) encode members of the GARP family of MYB-like TFs, predominantly expressed in the abaxial domains of lateral organs (Eshed et al. 2001). In *Arabidopsis*, genetic studies have revealed that mutations in individual *KAN* gene result in relatively mild leaf development defects (Kerstetter et al. 2001). However, the double mutant *kan1 kan2* exhibits reduced blade expansion and develops ectopic leaf-like outgrowths on the abaxial blade surface. Furthermore, the *kan1 kan2 kan3* triple mutants displays minimal blade expansion, yielding nearly cylindrical, adaxialized leaves with radialized stem vasculature (Eshed et al. 2001, 2004). Ectopic expression of individual *KAN* genes leads to profound abaxialization of lateral organs and disruption in vascular patterning (Kerstetter et al. 2001; Emery et al. 2003; Eshed et al. 2004).

Previous studies have identified a set of potential direct target genes of *KAN1*, revealing a significant enrichment of genes involved in the regulation of organ development and responsive to hormonal stimuli such as auxin, ABA and BR (Xie et al. 2015). Among these targets, the adaxial factor *ASYMMETRIC LEAVES2 (AS2)* is the most well-characterized target gene of KAN1. KAN1 represses *AS2* in abaxial tissue, leading to an adaxial phenotype (Wu et al. 2008). The identification of genes under dual regulation by the *REV*/*KAN1* module reveals distinct genetic networks for these antagonistic factors, enhancing our understanding of adaxial/abaxial regulatory network (Reinhart et al. 2013). These findings highlight critical role of *KAN* genes in determining leaf polarity, development and morphology.

Accessible chromatin regions (ACRs) represent putative cis-regulatory elements (CREs) targeted by TFs and are pivotal in transcriptional regulation, thereby influencing crucial agricultural traits. The Assay for Transposase Accessible Chromatin with high-throughput sequencing (ATAC-seq) has emerged as a valuable technology for profiling chromatin accessibility. Studies in wheat, *Arabidopsis*, rice, *Medicago* and tomato have identified CREs crucial for embryogenesis, leaf morphogenesis, and stress adaptation (Maher et al. 2018; Pei et al. 2023a, b). Recent work in maize has further demonstrated the utility of ATAC-seq in discovering functional CREs associated with development and trait formation (Liu et al. 2025).

In this study, we employed ATAC-seq to delineate the chromatin accessibility landscape during leaf heading in Chinese cabbage. Our analysis uncovered dynamic patterns of chromatin accessibility and identified distinct TFs associated with ACRs at each stage. Genes involved in adaxial-abaxial polarity and hormonal signaling pathways exhibited stage specific regulation, implicating their crucial roles in the heading process. Among these, two *BrKAN2* genes were identified as key TFs. Functional validation showed that *BrKAN2* mutations in Chinese cabbage and its overexpression in *Arabidopsis* significantly altered leaf morphology and adaxial-abaxial polarity. By integrating DAP-seq with ATAC-seq data, we defined a comprehensive set of direct BrKAN2.1 target genes, which were further validated through virus-induced gene silencing (VIGS) and electrophoretic mobility shift assays (EMSA). Furthermore, auxin responsiveness assays underscored the critical role of auxin signaling in regulating leaf heading. Collectively, our study provides a comprehensive transcriptional and regulatory framework for leaf head formation and offers a valuable resource for future genetic improvement of Chinese cabbage.

## Results

### A chromatin accessibility atlas of Chinese cabbage leaf heading

**Fig. 1:**
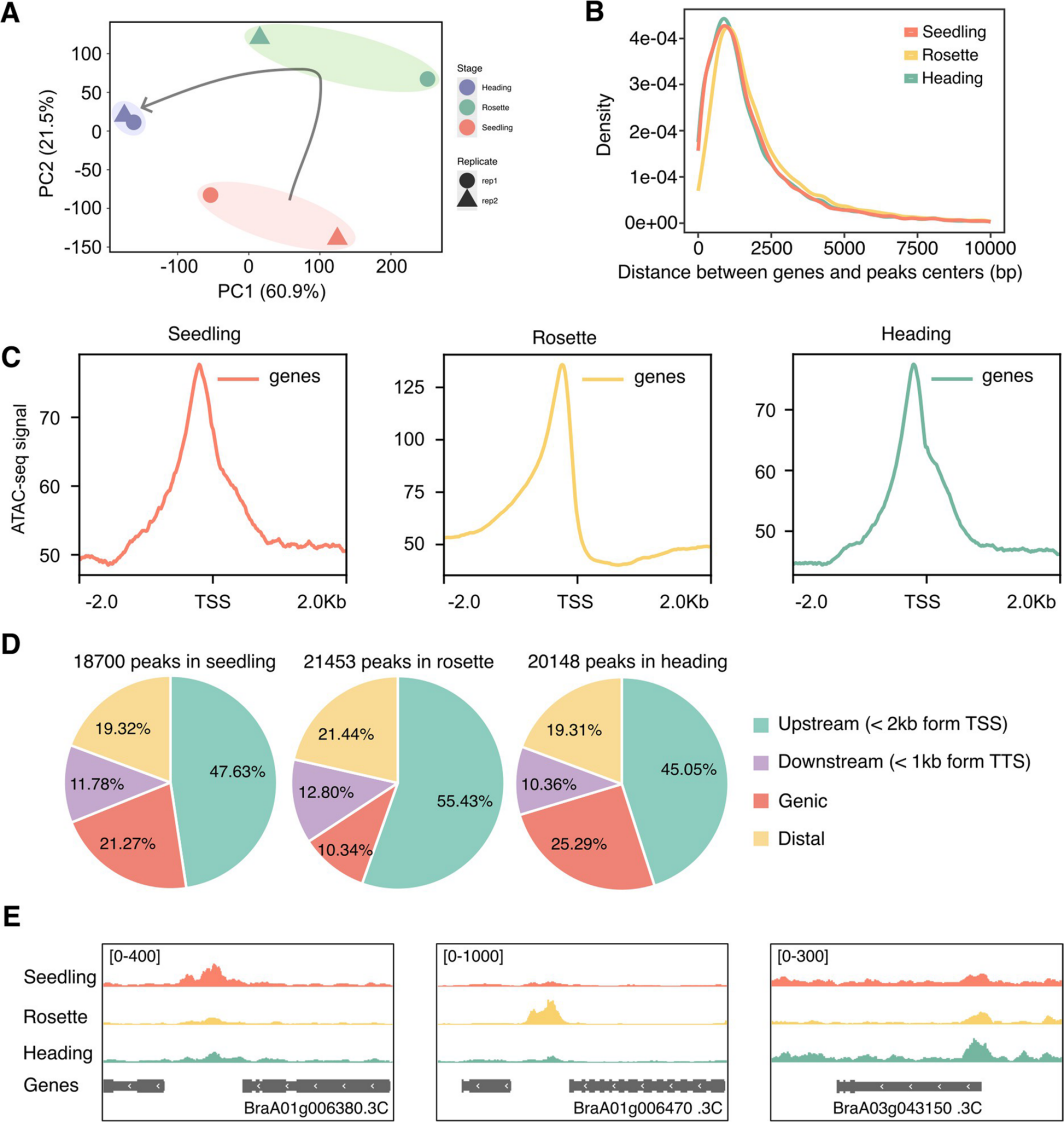
A chromatin accessibility atlas of Chinese cabbage leaf heading process. **A** Principal component analysis for ATAC-seq. **B** A frequency distribution of ACRs (accessible chromatin regions) and their distance to the nearest genes. **C** Profile of ATAC-seq along TSS (transcription start site) across three stages. **D** Genomic distribution of ACRs across the three developmental stages. **E** Integrative genomics viewer (IGV) showing the dynamic chromatin accessibility of representative genes

To characterize the chromatin accessibility landscape during Chinese cabbage leafy head formation, we performed ATAC-seq to map ACRs in leaves collected at three major developmental stages: seedling, rosette and heading stages, using two biological replicates per stage (Fig. S1A). The samples from three stages generated 88.0-285.9 million raw reads, and after filtering and deduplication, 18.9-40.0 million high-quality reads were retained for analysis (Table S1). Pearson correlation coefficients (*R* > 0.97) and principal component analysis (PCA) demonstrated a strong correlation between biological replicates and clear separation among developmental stages (Fig. 1A; Fig. S1B). Quality control further confirmed the integrity of all ACRs datasets (Table S2). Only ACRs consistently identified in both biological replicates were subjected to further analysis. Finally, a total of 18,700, 21,453 and 20,148 ACRs (peaks) were identified at the seedling, rosette, and heading stages, respectively.

Analysis of genomic distribution showed that most ACRs were located within 1 Kb of annotated genes (Fig. 1B). The identified ACRs exhibited prominent enrichment around transcriptional start sites (TSSs) (Fig. 1C). Most accessible regions are less than 1 Kb (Fig. S1C) and are concentrated near the telomere (Fig. S1D). Based on the distance between ACR centers and their nearest genes, we categorized the ACRs into genic ACRs (overlapping with gene bodies), proximal ACRs (within 2 Kb upstream of the transcription start site and within 1 Kb downstream of the transcriptional termination site) and distal ACRs (more than 2 Kb upstream of the transcriptional start site or within intergenic regions). The distribution pattern of ACR peaks across these three stages showed remarkable similarity, with the majority (45.1-55.4%) located within 2 Kb upstream of TSS. Around 19.3-21.4% of ACRs were located in the distal intergenic regions, and approximately 10.3-25.3% of ACRs were situated in the genic regions. Only 10.4-12.8% of ACRs were located within 1 Kb downstream of TTS. Collectively, the accessible regions across the three stages spanned 8.87 Mbp (2.48%), 13.70 Mbp (3.84%) and 11.29 Mbp (3.16%) of the Chinese cabbage genome (Fig. 1D). These results are consistent with previously reported ATAC-seq data in other crops (Li et al. 2025), supporting the robustness and reliability of our dataset.

### Chromatin accessibility is highly dynamic during Chinese cabbage leaf heading

**Fig. 2:**
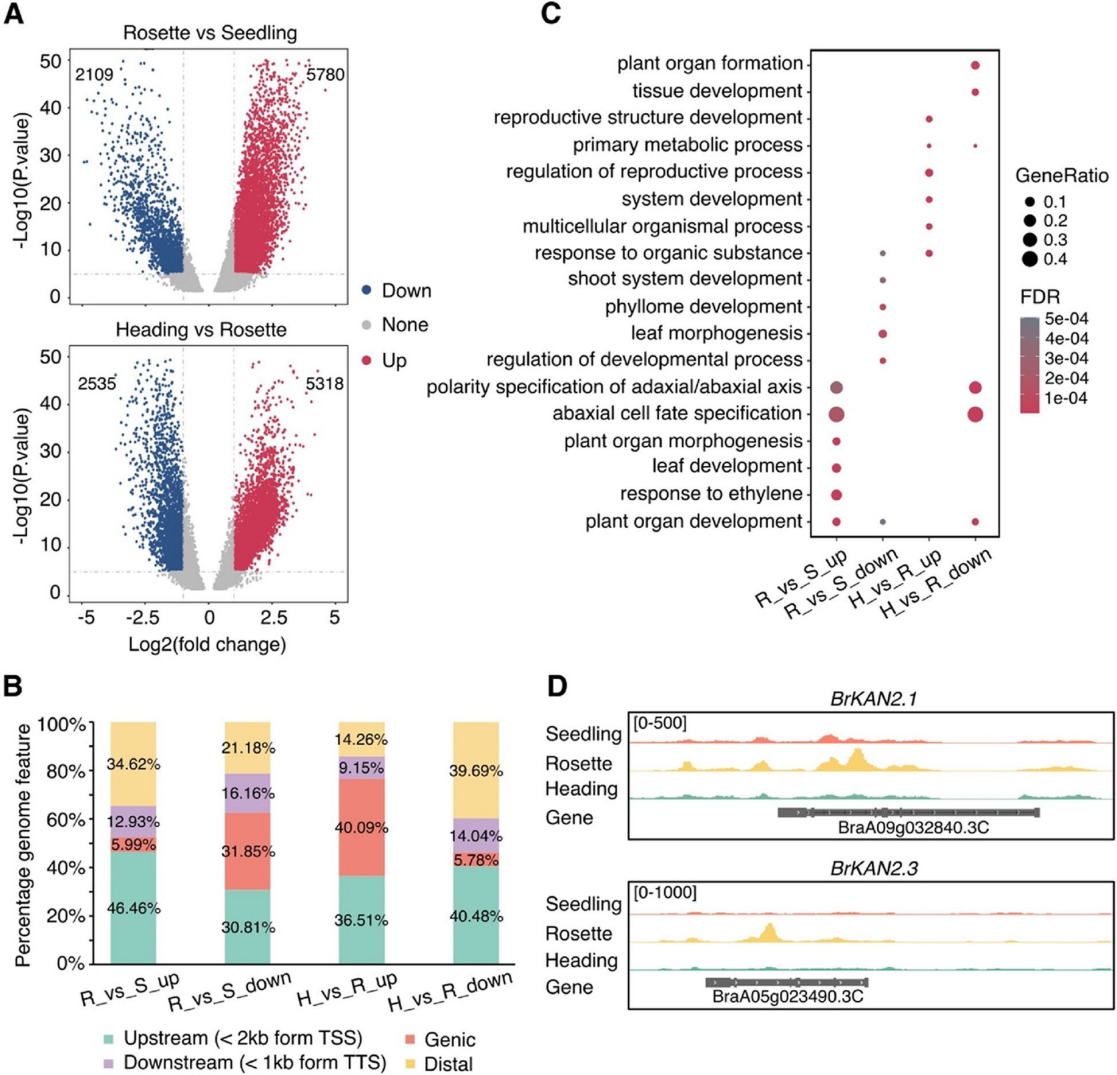
Chromatin accessibility dynamic change. **A** Volcano plots showing differential accessible chromatin regions (dACRs) between the rosette and seedling (top) and between the heading and rosette (bottom). The dACRs were defined with the following criteria (*p* < 1 × 10⁻^5^, |Log2(fold change) |≥ 1). **B** Distribution of 4 type dACRs on different genomic features including upstream, downstream, genic and distal region. **C** GO enrichment of TFs associated with dACRs under each comparison. **D** Dynamics chromatin accessibility of *BrKAN2* across the three developmental stages

We next performed pairwise comparisons of ACRs between consecutive developmental stages. A substantial number of ACRs showed significant changes in accessibility, categorized as differential ACRs (dACRs). These dACRs could be classified into two distinct types: downACRs, where accessibility peaks show significantly weaker signal at the later stage; while upACRs, where peaks show stronger signal at the later stage (Fig. 1E). In total, we identified 7,889 dACRs between the seedling and rosette stages, consisting of 2,109 downACRs and 5,780 upACRs. Similarly, between the rosette and heading stages, 7,853 dACRs were observed, comprising 2,535 downACRs and 5,318 upACRs, respectively (Fig. 2A). Notably, later developmental stages exhibited more ACRs compared to earlier stages.

Between the seedling and rosette stages, 65% of upACRs and 78% of downACRs were located in proximal and genic regions, while 35% and 22% were located in distal regions (Fig. 2B). Between the rosette and heading stages, 86% of upACRs and 60% of downACRs were similarly located in proximal and genic regions, with the remaining found in distal intergenic regions (Fig. 2B). The considerable disparities in chromatin accessibility between developmental stages underscore the notably dynamic nature of chromatin accessibility during the leaf heading process.

To elucidate the potential biological processes underlying the development of leaf heading, we performed Gene Ontology (GO) analysis to determine enriched categories of genes associated with dACRs. Our focus here was on the dACRs located in proximal and genic regions. Notably, genes associated with dACRs were highly enriched in transcriptional regulator activity during both the early (seedling and rosette stages) and later stage (rosette and heading stages) of leaf head formation (Fig. S2). Given the pivotal roles of TFs in gene expression regulation, we further conducted GO enrichment analysis on temporally TF genes associated with dACRs. Between the seedling and rosette stages, 487 upACRs associated TF genes were notably overrepresented in adaxial-abaxial polarity specification, abaxial cell fate specification, plant organ development and response to ethylene. Meanwhile, 87 downACRs associated TF genes showed enrichment in organ development, and leaf morphogenesis (Fig. 2C and S3). In contrast, between the rosette and heading stages, the 241 upACRs associated TF genes were enriched in reproductive process regulation, system development and primary metabolic process. Conversely, 236 downACRs associated TF genes were enriched in adaxial-abaxial specification, abaxial cell fate specification, and plant organ or tissue development (Fig. 2C and S3).

Interestingly, several genes involved in the adaxial-abaxial specification, and abaxial cell fate determination, such as *BrKAN2s*, *BrKAN1s*, *BrAS1*, *BrAS2*, *BrYAB2s* and *BrPHB*, were significantly enriched among upACRs-associated genes during the early developmental stages (seedling and rosette stages) (Table S3). Notably, some of those genes, including *BrKAN2s*, *BrKAN1s*, *BrAS1*, *BrYAB2s* and *BrPHB* were also enriched among downACRs-associated genes during the later developmental stage (rosette and heading stages) (Table S4 and Fig. 2D). These findings suggest that dynamic chromatin accessibility changes in genes regulating adaxial-abaxial specification, and abaxial identity may play crucial roles in controlling leafy head formation.

To corroborate these findings with transcriptome dynamics during leafy head formation, we examined the expression of adaxial-abaxial genes associated with dACRs by analyzing the published time-series RNA-seq data (Zhang et al. 2022a). It is noteworthy that our defined rosette stage in this study (approximately 50 days after sowing) for ATAC-seq corresponds to the leaf heading transition stage from the earlier RNA-seq work (Guo et al. 2022). As expected, a majority of these TFs genes were activated during the heading transition and heading stages. For instance, three *KAN* genes (two *BrKAN2s* and *BrKAN1.3*) were activated during the transition stage, while the expression of two *BrYAB* genes and *BrARF3.1* steadily increased, peaking at the heading mature stage (Fig. S4). However, two *BrKAN* genes (*BrKAN1.1* and *BrKAN1.2*) were activated at seedling or earlier rosette stage but inhibited during the transition stage and heading stage. Four genes (*BrAS1.3*, *BrAS2.2*, *BrPHB.1* and *BrJAG.1*) exhibited peak expression levels during the heading growth stage (Fig. S4). These findings suggest a potential association between alterations in chromatin accessibility of these leaf adaxial-abaxial TF genes and changes in their expression levels, thereby implicating their role in leafy heading process.

### Identification of TFs associated with leaf heading

Chromatin accessible regions potentially contain cis-acting elements bound by TFs that could regulate the expression of neighboring genes. To uncover the key TFs involved in the leaf heading process, we conducted motif enrichment analysis in dACRs. We identified 28 motifs in the rosette vs seedling comparison (R-vs-S) and 12 motifs in the heading vs rosette comparison (H-vs-R). Of these, 5 and 3 corresponding TFs were linked to leaf morphological development, respectively (Table 1, Table S5 and S6). As expected, the motifs for the key TFs TCP and SPL, known for their roles in leafy head formation, were identified. Furthermore, TFs from the GRF, MYB, KANADI, and ARF families were identified as potential key regulators of the leaf heading process (Table 1).

**Table 1:** Motifs identified from differentially accessible regions

TF	TF motif	*P*.value	% of Targets	% of Background
**Rosette vs Seedling**				
AT1G72010(TCP)		1e-193	19.68%	8.83%
GRF6(GRF)		1e-133	29.04%	17.69%
SPL7		1e-64	25.37%	17.67%
ARF18		1e-82	7.68%	3.19%
ARF3		1e-26	1.19%	0.31%
**Heading vs Rosette**				
MYB81(MYB)		1e-102	42.16%	30.06%
KAN4		1e-10	2.24%	1.33%
ARF1		1e-8	1.13%	0.56%

Remarkably, a member of KANADI TF (KAN4), belonging to GARP family of MYB-like TFs, was identified within R-vs-H enriched dACRs. Considering the conservation of predicted motifs and homologous TF proteins across different species, we focused on the homologous *KAN* genes, including three *BrKAN1* (*BrKAN1.1*, *BrKAN1.2* and *BrKAN1.3*) and two *BrKAN2* genes (*BrKAN2.1 *and* BrKAN2.3*). Notably, these genes exhibited distinct temporal changes in chromatin accessibility during development (Fig. 2D). Integrating our previously published RNA-seq data, we found that all these five *BrKAN* genes exhibited altered expression during leaf heading process, suggesting the potential involvement of these KAN TFs in leaf heading process (Fig. S4).

As previously mentioned, heading is a domestication trait, and our earlier investigations have emphasized the strong selection of the *BrKAN2.1* and *BrKAN2.3* genes in heading *B. rapa* (Liang et al. 2016; Cheng et al. 2016). In our examination of a resequencing dataset comprising 534 *B. rapa* accessions, which included 350 heading *B. rapa* (H-Br) accessions and 184 non-heading *B. rapa* (NH-Br) accessions (Cheng et al. 2016; Su et al. 2018; Cai et al. 2021), we further observed the selection signals of *BrKAN2.1* and *BrKAN2.3* for the heading trait. *BrKAN2.1* displayed three mutations in genomic region and two mutations in promoter region (Table S7, Fig. S5), while *BrKAN2.3* exhibited one mutation each in the genomic and promoter regions (Table S7, Fig. S6), all showing significant biased distribution between the H-Br and NH-Br groups. These findings strongly associate H-Br alleles of *BrKAN2.1* and *BrKAN2.3* with the leaf heading trait. Taking these results into account, we speculate that two BrKAN2 TFs may play a role in leaf heading of Chinese cabbage.

### Two BrKAN2 homeologs control leaf morphology

**Fig. 3:**
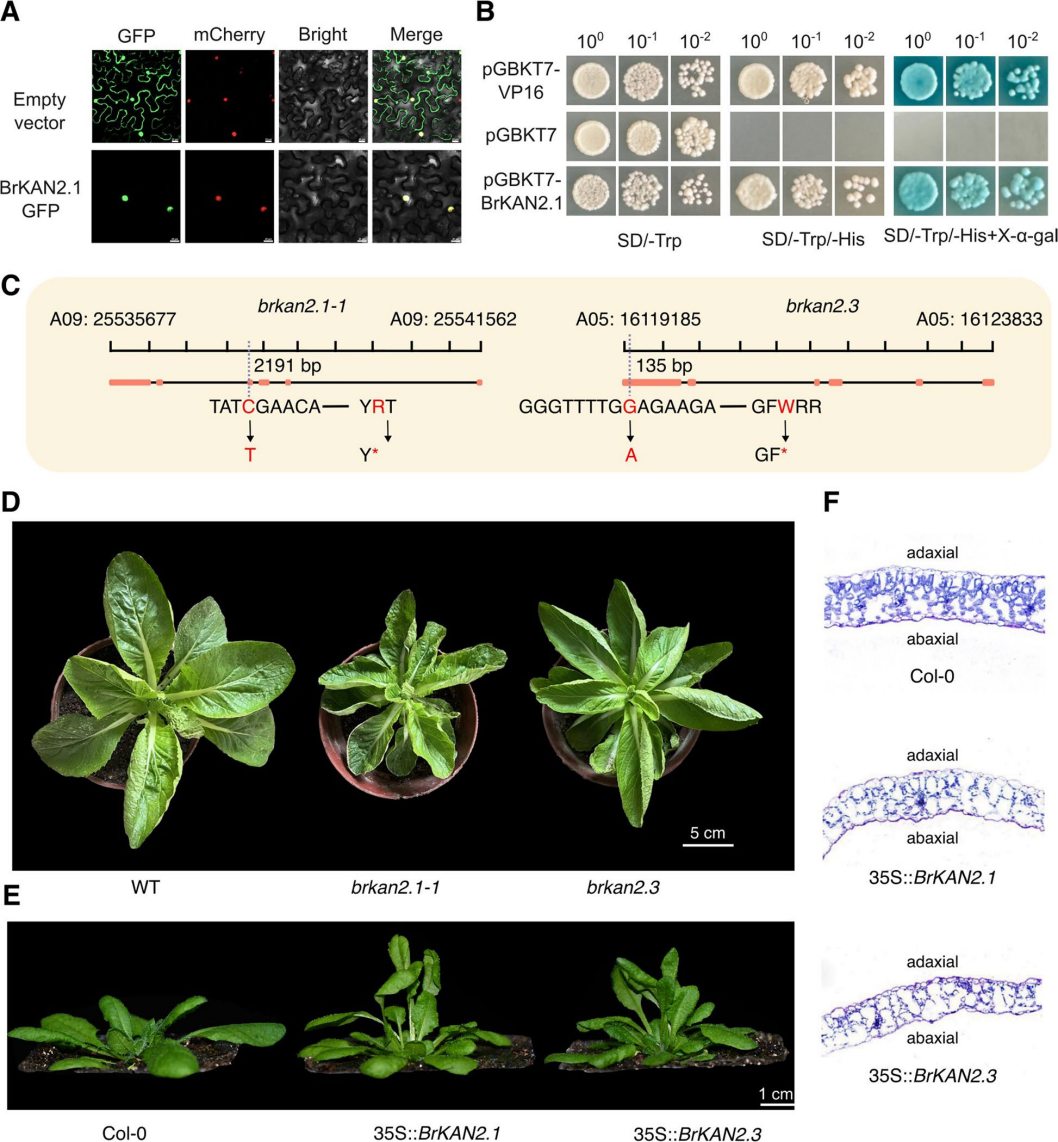
BrKAN2 regulates leaf morphology in both Chinese cabbage and *Arabidopsis*. **A** Subcellular localization of BrKAN2.1 in tobacco epidermal cells. Fluorescence signals from free GFP and the nuclear localization marker mCherry (controls), as well as the BrKAN2.1-GFP fusion protein, are shown. Scale bar, 20 μm. **B** BrKAN2.1 exhibiting transactivation activity in a yeast assay system. **C** Schematic diagram of mutation sites in *BrKAN2.1* and *BrKAN2.3* genes. **D** Representative leaf images of wild-type Chinese cabbage and *brkan2* mutants. **E** Representative leaf images of Col-0 wild-type and *BrKAN2* overexpressing *Arabidopsis* plants. **F** Altered leaf cellular structure in *BrKAN2* overexpression lines compared to Col-0 wild-type

The two predicted BrKAN2 proteins shared a high identity of 91.7%, displaying substantial similarity (91-92%) to the sequence of *Arabidopsis* AtKAN2 (Table S8). Notably, both of these predicted BrKAN2 proteins harbored a highly conserved MYB domain (Fig. S7). Phylogenetic analysis revealed that KAN2 exhibits a relatively close relationship with KAN1 (Fig. S8). Subcellular localization assay using the 35S::BrKAN2.1-GFP construct in *Nicotiana benthamiana* epidermal cells revealed that BrKAN2.1 is localized in the nucleus (Fig. 3A). A yeast transactivation assay confirmed that BrKAN2.1 possesses transcriptional activation activity (Fig. 3B).

To examine the role of BrKAN2 genes in the regulation of leaf morphology in Chinese cabbage, we screened a Chinese cabbage mutant library and identified two allelic *brkan2.1* mutants, harboring premature termination mutations at positions 2,191 bp and 292 bp, respectively (Fig. 3C and S9A). Additionally, a *brkan2.3* mutant with a premature stop coding at 135 bp were also identified (Fig. 3C). Both allelic *brkan2.1* mutants and the *brkan2.3* mutant exhibited thinner leaves and pronounced inward curling compared to wild-type plants (Fig. 3D and S9B). Morphological measurements further revealed distinct phenotypic differences: the wild type reached average height of 14 cm, whereas *brkan2.1* and *brkan2.3* mutants were shorter, at 7.9 cm and 9.6 cm, respectively. The leaf length-to-width ratio was 1.71 for the wild-type, compared to 1.79 and 2.34 for the mutants, while the average leaf angle was 36° for the wild-type, versus 62° and 70° for the mutants (Table S9). These findings underscore the involvement of *BrKAN2* genes in leaf morphology formation and structural stability during leafy head development in Chinese cabbage.

In addition, the full-length cDNA of each *BrKAN2*, under the control of the CaMV 35S promoter, was transformed into *Arabidopsis* Col-0. The gain-of-function lines overexpressing either *BrKAN2* gene displayed similar pleiotropic leaf phenotypes (Fig. 3E), including a significant increase in rosette leaf number, reduced leaf length and width, and an elevated petiole-to-leaf ratio (Fig. S9C-F). Notably, these lines also exhibited epigastric leaf growth with downward curling and reduced leaf angles, demonstrating the role of *BrKAN2* genes in leaf morphology regulation. Anatomical analysis revealed that the transgenic leaves lacked compact palisade parenchyma and instead contained spongy mesophyll on both sides (Fig. 3F), suggesting that BrKAN2.1 and BrKAN2.3 disrupt adaxial-abaxial leaf patterning.

### Genome-wide identification of BrKAN2 target genes

**Fig. 4:**
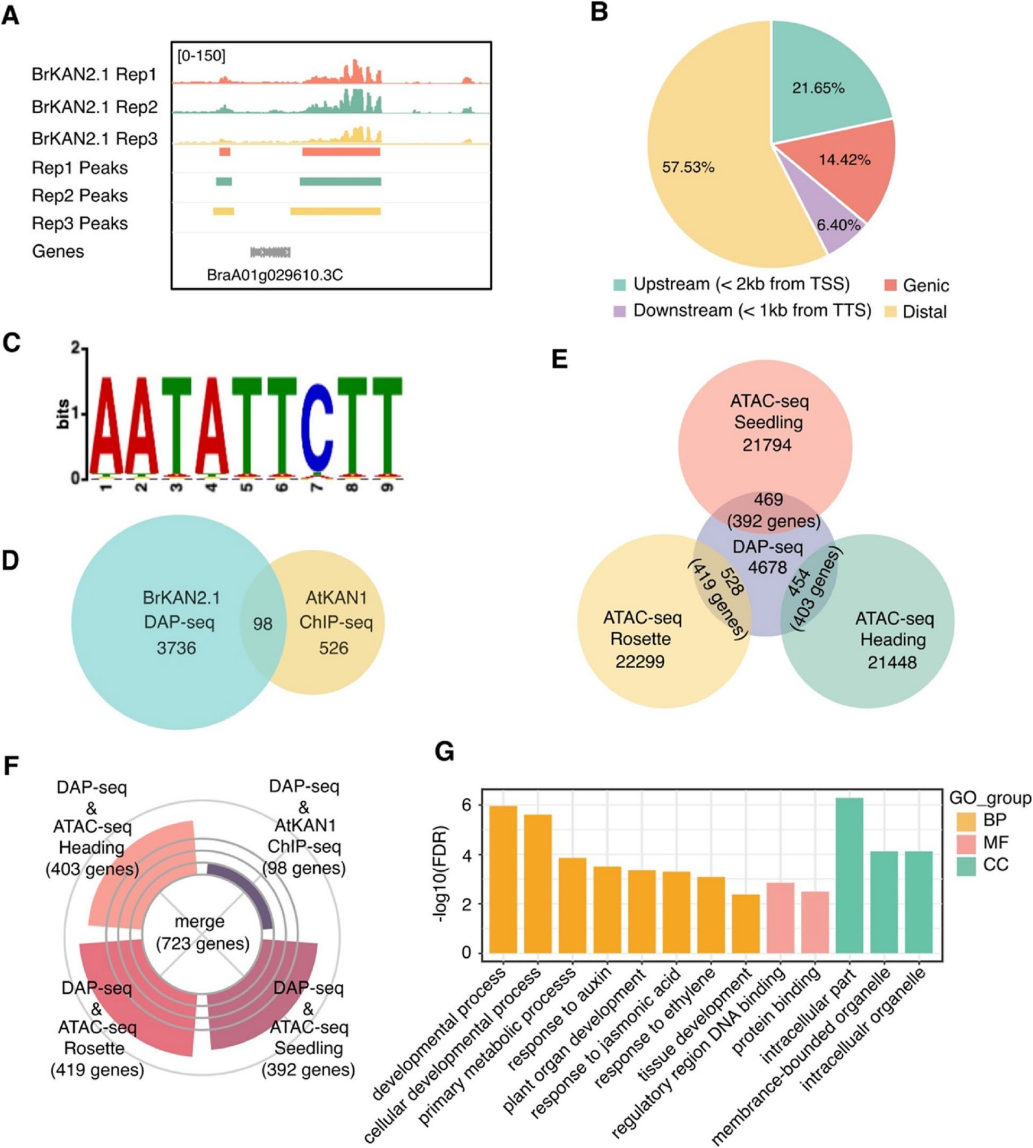
BrKAN2.1 DAP-seq analysis and GO enrichment of its potential target genes. **A** Sample screen shot of genome browser with DAP-seq peaks for BrKAN2.1. **B** Distribution of BrKAN2.1 binding sites on different genomic features. **C** The identified binding motifs of BrKAN2.1 protein by MEME-ChIP. **D** Venn diagram showing overlap between BrKAN2.1 and AtKAN1 target genes. **E** Venn diagram showing overlap between BrKAN2.1 binding sites and ACRs regions at the three developmental stages. **F** Nightingale rose diagram showing the overlap between BrKAN2.1 target genes and stage specific ACRs associated genes, and between BrKAN2.1 and AtKAN1 target genes, respectively. **G** Gene ontology (GO) enrichment analysis of intersecting genes from ATAC-seq, DAP-seq, and ChIP-seq datasets

To elucidate the potential molecular mechanism underlying BrKAN2’s involvement in the regulation of leaf morphology, we employed DAP-seq to delineate the genome-wide binding profiles of BrKAN2.1. The DAP-seq assay was performed with three independent biological replicates, which exhibited a high Pearson correlation coefficient of 0.98-0.99 (Fig. S10A). A representative view from the IGV illustrates several BrKAN2.1 binding sites (Fig. 4A) and the genome wide binding profile further underscores the high reproducibility (Fig. S10B). Binding signals were predominantly enriched around the TSS regions (Fig. S10C). A total of 4,678 high-confidence BrKAN2.1 binding peaks were identified across three independent DAP-seq replicates.

A comprehensive examination of the genome wide distribution unveiled about 57.53% of these peaks were located in distal region of putative target genes, with about 21.65% residing in the upstream promoter regions of putative target genes. Binding within the coding sequence or downstream regions accounted for 14.42% and 6.4% of the peaks, respectively (Fig. 4B). Utilizing the 4,678 identified peaks, we pinpointed a total of 3,736 putative genes bound by BrKAN2.1, each harboring one or more binding peaks. Additionally, we used MEME to uncover the cis-regulatory motif crucial for BrKAN2.1 binding. A conserved binding motif AATATTCTT present in 3,527 peaks (75.4%). Notably, the TATTC sequence corresponds to the inner core of the inverted palindromic motif VGAATAW, a well-established KAN1-binding motif in *Arabidopsis* (Fig. 4C).

To explore the functional conservation of KANADI proteins, we compared the putative BrKAN2 target genes identified in this study with published target genes of AtKAN1 in *Arabidopsis*, which were derived from ChIP-seq datasets (Xie et al. 2015). This analysis identified 98 conserved target genes shared between BrKAN2 in Chinese cabbage and AtKAN1 in *Arabidopsis* (Fig. 4D). Among these 98 genes, some are known to have a role in auxin biosynthesis and signaling such as *PIN1*, *SAUR52*, *IAA2*, and *ARF4*. Interestingly, *SAW1* and *SAW2* were also identified, which belong to BEL gene family. Several members of *BEL* gene family are established targets of KAN1 in *Arabidopsis* (Xie et al. 2015). Besides, *SAW1* and *SAW2* are known to be involved in leaf heading in Lettuce (An et al. 2022). These results highlight the relative conservation of the cis-regulatory motif and downstream target genes regulated by different members of the KAN TFs across different species. Furthermore, they lend support to the validation of our screening for BrKAN2 target.

As previously delineated, ACRs represent nucleosome-free regions occupied by various TFs. To refine our catalog of candidate direct target genes of BrKAN2.1 implicated in leaf heading, we subsequently integrated DAP-seq data with three temporal ATAC-seq datasets. Across the respective datasets, 469, 528 and 454 of the total ACRs exhibited overlap with BrKAN2.1 binding peaks (Fig. 4E). These regions not only exhibited open chromatin during at least one stage but also were potentially bound by BrKAN2.1. We further identified 657 genes closest to these regions. Integrating these 657 genes with the aforementioned 98 genes, we ultimately obtained a set of 723 genes as the putative target genes of BrKAN2 after removing redundancies (Fig. 4F). These 723 genes correspond to 646 homologous genes in *Arabidopsis*.

The GO enrichment analysis of these BrKAN2.1 bound genes revealed significant enrichment in development process, primary metabolic process, and the response to hormones such as auxin, jasmonic acid, and ethylene (Fig. 4G, Table 2, Table S10, S11 and S12). These findings indicated a potential crucial role for BrKAN2 in modulating hormone signaling pathways during Chinese cabbage leaf heading. Of 723 genes identified as putative BrKAN2.1 targets, 25 are involved in auxin response. Table 2 shows a subset of BrKAN2.1 target genes encoding proteins involved in auxin biosynthesis as well as in auxin signaling. This set of genes includes *PIN1*, Aux/IAA genes (*IAA2*), three SAUR-LIKE genes (*SAUR50*, *SAUR53* and *SAUR72*), *WAG2*, and *AXR6*. Furthermore, four AUXIN RESPONSE FACTOR genes, *ARF4*, *ARF5* and *ARF6*, were identified.

**Table 2:** Target gene of auxin response pathway

*B. rapa*_ID	*Ath*_ID	Annotation	*B. rapa*_ID	*Ath*_ID	Annotation
*BraA06g029000.3C*	*AT5G65510*	*AIL7*	*BraA01g042490.3C*	*AT3G05630*	*PDLZ2*
*BraA10g014170.3C*	*AT5G56290*	*PEX5*	*BraA03g059020.3C*	*AT4G34760*	*SAUR50*
*BraA02g010280.3C*	*AT5G60450*	*ARF4*	*BraA07g016220.3C*	*AT1G19840*	*SAUR53*
*BraA07g016200.3C*	*AT1G19850*	*ARF5*	*BraA05g034250.3C*	*AT3G12830*	*SAUR72*
*BraA09g034710.3C*	*AT1G30330*	*ARF6*	*BraA08g027360.3C*	*AT1G21410*	*SKP2A*
*BraA08g023340.3C*	*AT1G30330*	*ARF6*	*BraA05g003020.3C*	*AT2G42580*	*TTL3*
*BraA04g014760.3C*	*AT5G37770*	*CML24*	*BraA07g039050.3C*	*AT1G75500*	*WAT1*
*BraA06g004800.3C*	*AT4G02570*	*AXR6*	*BraA05g032690.3C*	*AT3G14370*	*WAG2*
*BraA08g034980.3C*	*AT1G04250*	*AXR3*	*BraA07g016630.3C*	*AT5G67300*	*MYB44*
*BraA03g009960.3C*	*AT5G20990*	*CHL6*	*BraA02g021850.3C*	*AT1G73590*	*PIN1*
*BraA04g020930.3C*	*AT5G65940*	*CHY1*	*BraA08g010550.3C*	*AT4G14430*	*ECI2*
*BraA08g011080.3C*	*AT1G28130*	*GH3.17*	*BraA08g000170.3C*	*AT1G56010*	*NAC021*
*BraA03g040660.3C*	*AT3G23030*	*IAA2*			

**Fig. 5:**
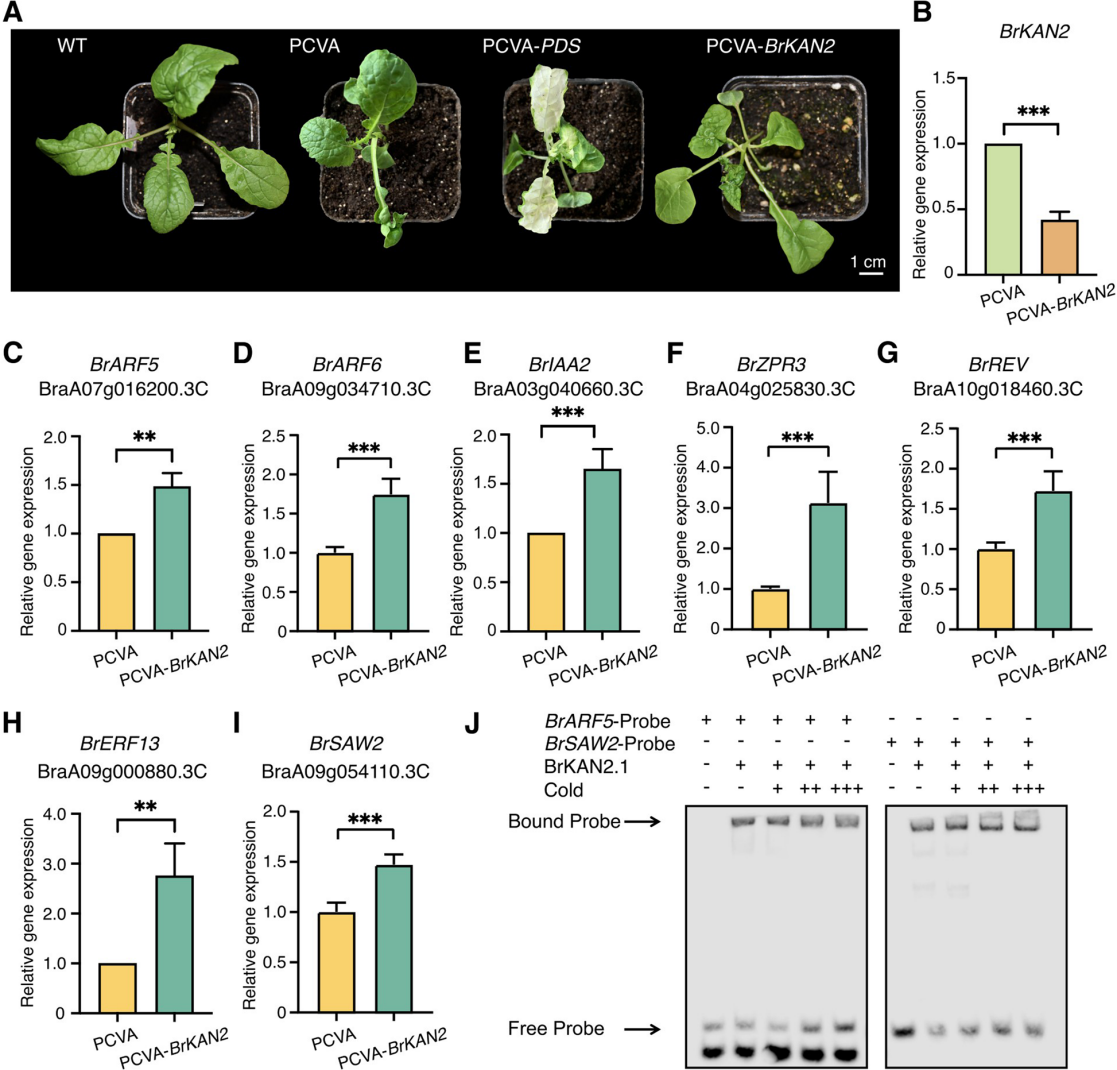
VIGS experiments validated the target genes of BrKAN2. **A** Phenotypes of *BrKAN2* silenced plants following VIGS. The WT, PCVA, PCVA-PDS, and PCVA-BrKAN2 denote mock-infiltrated plants, and those infiltrated with PCVA empty vector, PCVA-*PDS*, and PCVA-*BrKAN2*, respectively. **B** Relative expression levels of *BrKAN2* in PCVA-*BrKAN2* and PCVA plants. The error bars represent the standard deviations of three independent biological repeats. The asterisks represent significant differences via T-tests (*p ≤ 0.05; **p ≤ 0.01; and ***p ≤ 0.001).** C**-**I** Relative expression levels of BrKAN2.1 target genes in PCVA-*BrKAN2* and PCVA plants. **J** Specific binding of BrKAN2.1 to conserved motifs within the regulatory regions of *BrARF5* and *BrSAW2*

To verify the potential target genes, the *BrKAN2* gene was silenced using VIGS technology (Fig. 5A). The expression level of *BrKAN2.1* was significantly reduced in VIGS treated plants compared to the control (Fig. 5B). The expression levels of fourteen selected genes with the identified motif in their proximal promoters exhibited significant changes in VIGS plants relative to controls. These include three auxin-related genes (*BrARF5*, *BrARF6* and *BrIAA2*) (Fig. 5C-E), two abaxial-adaxial polarity-regulated genes (*BrZPR3.2* and *BrREV.1*) (Fig. 5F, G), one ethylene-related gene (*BrERF13*) (Fig. 5H), one leaf development gene (*BrSAW2*) (Fig. 5I) and three *BRX* genes (*BrBRX.1*, *BrBRX.2* and *BrBRX.3*) (Fig. S11), indicating that they are targets of BrKAN2. Besides, four other genes including *BrKAN1*, *BrKAN3*, *BrIPT7* and *BrWAG2* (Fig. S11), exhibited slightly reduced expression in VIGS plants compared to controls. EMSA was performed to further verify BrKAN2.1 bound to the target genes *BrARF5* and *BrSAW2* (Fig. 5J). Collectively, our results demonstrate that BrKAN2.1 regulates the expression of its target genes through direct binding.

### BrKAN2 mediated regulation of leaf heading involves auxin signaling

**Fig. 6:**
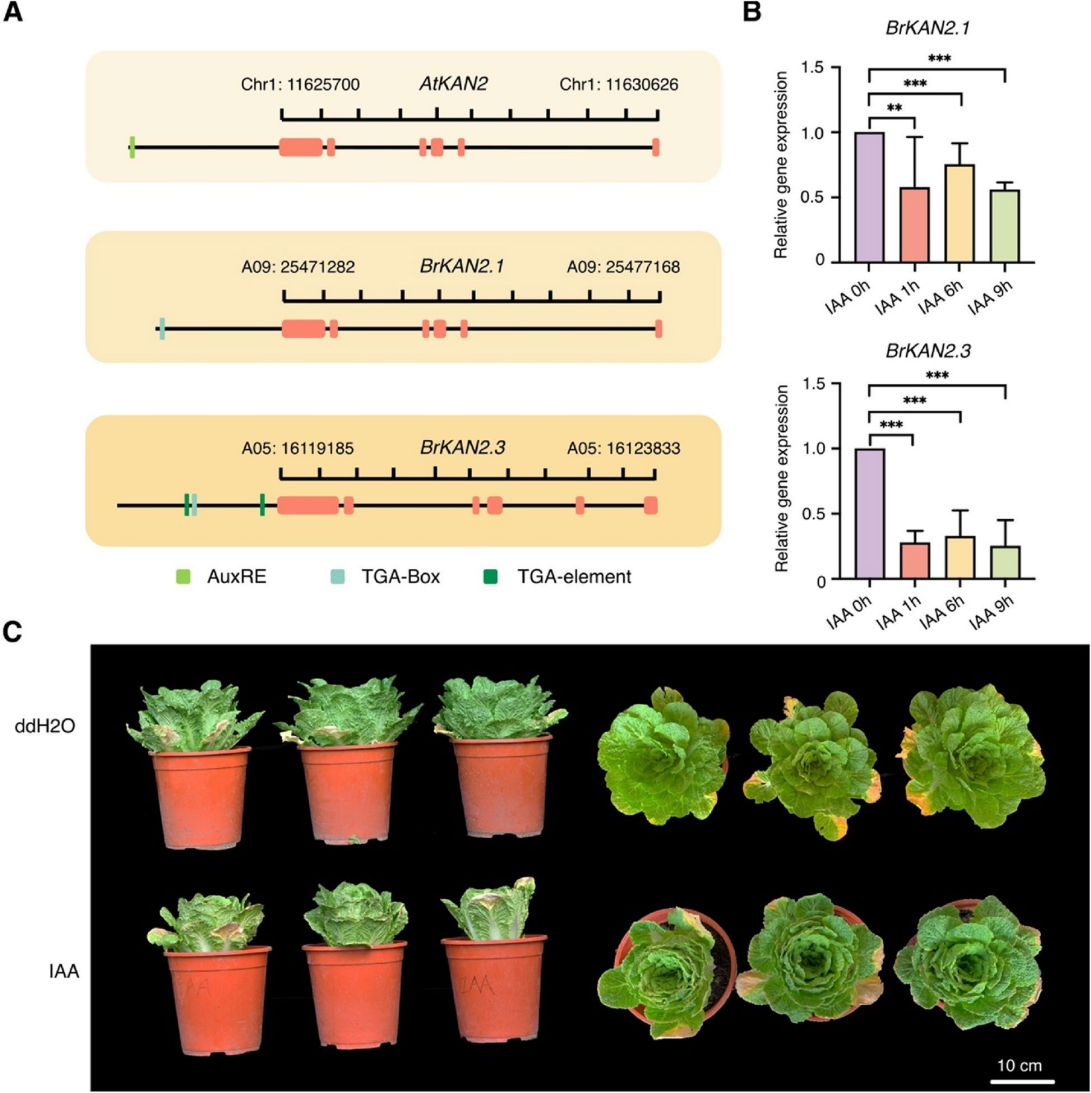
BrKAN2 mediated regulation of leaf heading involves auxin signaling. **A** Predicted auxin response elements in the promoter regions of *AtKAN2*, *BrKAN2.1* and *BrKAN2.3* genes. **B** Relative expression of *BrKAN2* genes under IAA treatments. The error bars represent standard deviations of three independent biological repeats. Asterisks represent significant differences via T-tests (*p ≤ 0.05; **p ≤ 0.01; and ***p ≤ 0.001). **C** Leafy head phenotypes of Chinese cabbage under IAA treatment. The images were captured at the two months growth stage of the Chinese cabbage

We further analyzed the promoter sequences of the two *BrKAN2* genes, specifically 2 Kb upstream of the transcription start site. Multiple cis-acting elements related to plant hormone responses (auxin, GAs and JA) were identified (Fig. 6A, Table S13). This finding suggested that plant hormones may regulate the expression of *BrKAN2* genes. Considering BrKAN2 also binds to several auxin related genes (*BrARF5*, *BrARF6* and *BrIAA2*), we assess the responsiveness of the two *BrKAN2* genes to auxin. RT-qPCR analysis was conducted to measure relative expression of *BrKAN2* levels upon IAA treatment. The expression levels of *BrKAN2.1* and *BrKAN2.3* were strongly inhibited following IAA treatment (Fig. 6B).

Additionally, to explore the potential involvement of *BrKAN2* genes in auxin-mediated regulation of leaf heading, we performed an IAA treatment assay during the rosette stages of Chinese cabbage, repeating the treatment every two days for one month. We found that IAA treatment induced the leaf heading, resulting in earlier head formation and a more compact architecture compared to the control (Fig. 6C). Taken together, these results suggest that the *BrKAN2* genes in Chinese cabbage may play a role in regulating the leaf heading process through their involvement in auxin signaling pathway.

## Discussion

**Fig. 7:**
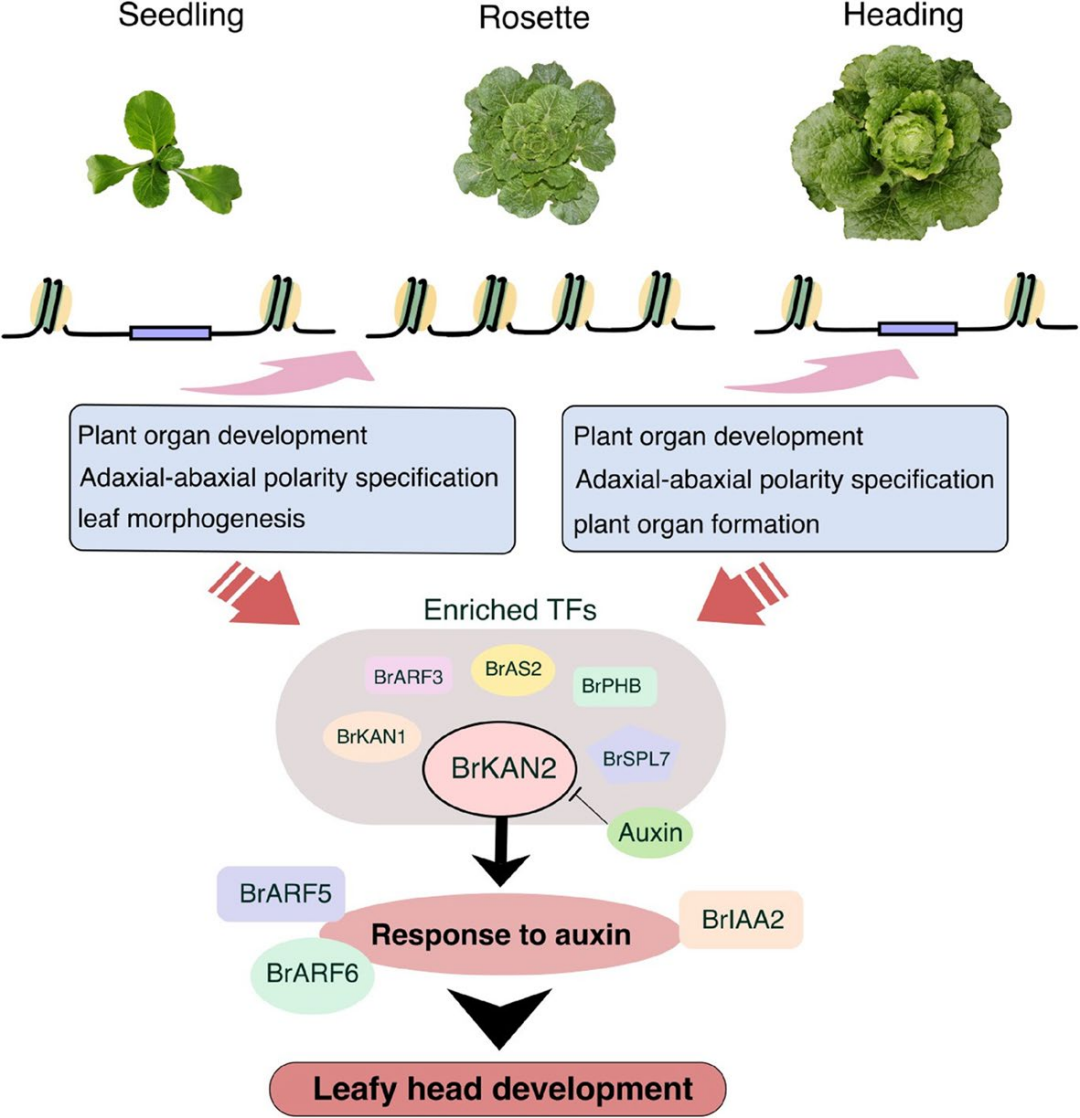
Proposed regulatory model of leafy head formation in Chinese cabbage. During leafy head development in Chinese cabbage, dynamic changes in chromatin accessibility coordinate key developmental processes such as organ development, leaf morphogenesis, and adaxial-abaxial polarity establishment. The transcription factor BrKAN2 plays a crucial role in this process by directly binding to target genes such as *BrARF5*, thereby participating in auxin-mediated hormonal signaling pathways to regulate leafy head formation

Leaf heading in Chinese cabbage is a complex developmental process involving significant changes in leaf morphology and gene expression. Using ATAC-seq, we uncovered dynamic chromatin accessibility patterns throughout this process, identifying key TFs and genes involved in leaf heading. Notably, *BrKAN2* genes emerged as pivotal regulators. Functional characterization of *BrKAN2* through mutant analysis in Chinese cabbage and overexpression in *Arabidopsis* revealed significant alterations in leaf morphology and adaxial-abaxial polarity. Through DAP-seq and ATAC-seq, we identified a set of potential BrKAN2 target genes enriched in organ development and hormone-related pathways, underscoring the pivotal role of BrKAN2 in coordinating auxin mediated regulation during leaf heading (Fig. 7). These results provide valuable insights into the molecular mechanisms governing leafy head formation in Chinese cabbage and establish a foundation for future functional studies aimed at further understanding and improving leaf heading.

We observed a significant enrichment of ethylene related genes, including *BrERFs*, *BrEINs* and *BrEILs*, among upACRs associated genes during the transition from seedling to rosette stage. Our previous study documented a pronounced activation of ethylene pathway genes during this stage, with genes such as *BrERFs*, *BrEINs*, *BrEBFs* and *BrEILs* showing enhanced expression level (Zhang et al. 2022a). These observations suggest a potential association between changes in chromatin accessibility of ethylene pathway genes and their expression levels, thereby implicating the involvement of the ethylene pathway in leaf heading. Consistent with this, ethylene treatment increased leaf angle, promoting a more compact and erect leaf architecture, confirming the role of ethylene in leaf heading (Zhang et al. 2022a).

Additionally, genes involved in adaxial-abaxial specification, particularly those related to abaxial cell fate determination, were enriched among both upACRs associated genes during the transition from seedling to rosette stage and downACRs associated genes during the shift from rosette and heading stage. Notably, certain genes implicated in adaxial-abaxial specification, such as two *BrKAN2* genes, three *BrKAN1* genes, *BrAS1.3*, *BrAS2.2*, *BrPHB.1* and *BrYAB2.2*, *BrYAB2.3* displayed a pattern of increasing ACRs from seedling to rosette stage, followed by a decrease from the rosette to heading stage. As expected, most of these adaxial-abaxial genes were activated during the heading transition and heading stages, suggesting their involvement in leafy head development.

One of the goals of this study was to identify key TFs associated with chromatin accessibility changes responsible for leafy head development. Motif enrichment analysis of dACRs revealed motifs corresponding to key TFs, such as TCP and SPL, which are known for their roles in leaf head formation. Specifically, TCP4 and SPL9 have been show to play important roles in leaf heading of Chinese cabbage (Mao et al. 2014; Wang et al. 2014). Furthermore, TFs from KAN, ARF, PCF, GRF, and MYB families were identified as potential regulators of the leaf heading process. KAN1-4 and ARF3/ARF4 are well-known for their involvement in leaf adaxial-abaxial specification. Notably, two *BrKAN2* and *BrARF3.1* genes have undergone strong selection during the evolution of heading *B. rapa* (Cheng et al. 2016). Moreover, changes in the chromatin accessibility of these genes are correlated with alterations in their expression levels during leafy head formation (Fig. S3). These findings strongly suggest the involvement of two *BrKAN2* and *BrARF3.1* genes in the leaf heading process. The Chinese cabbage *brkan* mutant exhibits reduced plant height and upward-curling leaves. In contrast, overexpression of these two *BrKAN2s* in *Arabidopsis* leads to downward-curling leaves, reduced leaf angle, and disruption of leaf adaxial-abaxial polarity. Given that leaf curling and upright growth are crucial for head formation, *BrKAN2* genes may be involved in this process. Similar to these findings, previous studies showed overexpression of *AtKAN1* and *AtKAN2* in *Arabidopsis* leads to abnormal cotyledon development, producing elongated and downward-curling cotyledons (Eshed et al. 2001; Wu et al. 2008). These consistent phenotypes across studies not only validate our findings but also underscore the conserved role of KANADI genes in regulating lateral organ development. Unfortunately, we were unable to obtain mutant plants at the heading stage, because the EMS mutants exhibited weak growth and sterility. In the future, CRISPR/Cas9 mutants can further validate the phenotype and refine our understanding of BrKAN2 function.

*KAN1* is well known for its role in leaf, shoot, and ovule patterning. Previous studies integrating ChIP-seq, Tilling Array gene expression profiling, and microarray have identified its putative direct target genes, including those involved in patterning and growth-promoting pathways, auxin biosynthesis and signaling (Xie et al. 2015). In this study, we employed DAP-seq to identify direct targets of BrKAN2.1. Our analysis suggests that the AATATTCTT motif may function as a cis-regulatory element recognized by BrKAN2, similar to the VGAATAW motif for KAN1 binding in *Arabidopsis*. Comparative analysis with *Arabidopsis* KAN1 ChIP-seq data revealed an overlap of 98 genes, which are homologous with 87 potentially direct targets of KAN1, indicating shared targets between BrKAN2 and AtKAN1. This suggests a relative conservation of cis-regulatory motifs and downstream target genes across species, supporting the validity of our BrKAN2 target screening.

To refine the list of candidate direct targets, we integrated DAP-seq data and three ATAC-seq datasets identifying a total of 657 genes. Combining these with the aforementioned 98 genes, we obtained a set of 723 putative target genes of BrKAN2.1 after removing redundancies, which are homologous with 646 genes in *Arabidopsis*. Among these 723 genes, several downstream targets are involved in organ development, shoot patterning, and hormonal pathways, including auxin, ethylene and jasmonic acid biosynthesis and signaling. This suggest that BrKAN2 regulates leaf heading by orchestrating versatile molecular processes. Enrichment analysis indicated a significant presence of auxin related genes. We experimentally confirmed that three auxin related genes were up regulated in *BrKAN2*-VIGS plants. And IAA treatment significantly suppressed the expression of *BrKAN2.1* and *BrKAN2.3*, leading to earlier head formation and a more compact leaf architecture in Chinese cabbage. These findings support previous research that overexpression of AUXs which regulate auxin concentration, induces leafy head formation in Chinese cabbage (He et al. 2000). While we have demonstrated the contribution of the auxin pathway to leafy head development, other hormone pathways may also play significant roles. Our BrKAN2 candidate targets analysis revealed enrichment of ethylene related and jasmonic acid related genes. Our previous study documented a pronounced activation of ethylene related genes including *ETR2*, *ERS2s*, *EIN3s*, *ERFs* and *ACSs* during the heading transition stage. Ethylene treatment resulted in more erect leaves and a compact architecture, suggesting its role in leafy head growth (Zhang et al. 2022a). Noteworthy, in our study, *ERFs* are upregulated by BrKAN2, implying that BrKAN2 may influence leafy head development by modulating ethylene pathway. However, jasmonic acid treatment had no significant effect on the formation of leafy heads in our study. Therefore, future research could focus on clarifying the precise role of the BrKAN2 in response to ethylene in regulating leafy head development.

Our study identified *SAW2* genes as the potential direct target of BrKAN2. A recent study identified *SAWTOOTH1*(*LsSAW1*) as a gene controlling leafy head formation in lettuce (An et al. 2022). Loss of function mutations in *LsSAW1* lead to leafy head formation by downregulating adaxial genes and the upregulating abaxial genes. Moreover, *LsSAW1* and *LsSAW2* exhibit similar functions in the development of leafy head (An et al. 2022). In *Arabidopsis*, *SAW1* and *SAW2* redundantly regulate leaf serration (Kumar et al. 2007). The fact that *SAW2* gene is highly regulated by BrKAN2, suggesting its involvement in leafy head formation of Chinese cabbage and providing a guidance for future experiments to explore the function of *SAW2*.

Additionally, the *BrBRX* gene was isolated as a putative target of BrKAN2.1 through DAP-seq, and its expression was upregulated in *BrKAN2*-VIGS plants. Our previous study indicated that *BrBRX* might play a role in leaf heading, as it was under strong selection during heading *B. rapa* (Cheng et al. 2016). Overexpression of all three *BrBRX* in *Arabidopsis* led to phenotypes resembling those of *BrKAN2* overexpressing lines, including increased leaf number and backward curling (Zhang et al. 2021). As *BrBRX* expression are also auxin-responsive (Zhang et al. 2021), these results suggest that BrBRX and BrKAN2 may act within a shared regulatory pathway controlling leaf morphology. However, further experimental validation is required.

The predicted target genes of BrKAN2 identified in this study provide a valuable resource for further functional studies. With advances in transgenic and gene-editing technologies, there targets can be experimentally validated, shedding light on the mechanisms by which BrKAN2 regulates leafy head formation in Chinese cabbage.

## Materials and methods

### Plant growth

*B. rapa* (Chinese cabbage cv Chiifu-401–42) was grown in the field at Chinese Academy of Agricultural Sciences (Beijing, China) until leafy heads formed, and used ATAC-seq, *BrKAN2* cloning, and auxin treatment. *B. rapa* (Sarson R-O-18) was cultured on MS medium and then plant in the greenhouse (25 $^\circ{\rm C}$ with 16 h photoperiod) for VIGS.

*Arabidopsis* (Col-0) were grown on MS medium to four-leaf stage and then transferred to a climate chamber for overexpression studies (25 $^\circ{\rm C}$ with 16 h photoperiod).

### ATAC-seq library preparation

ATAC-seq was described by Lu et al. (2017). For each sample, approximately 1 g flash-frozen leaves were chopped with a razor blade in 1 mL ice-prechilled lysis buffer (15 mM Tris–HCl pH 7.5, 20 mM NaCl, 80 mM KCl, 0.5 mM spermine, 5 mM 2-Mercaptoethanol, 0.2% Triton X-100) and filtered twice through a 40 μm mesh. Nuclei were stained with DAPI and analyzed by a flow cytometer. Nuclei pellets were obtained after centrifuged and washed with Tris-Mg buffer (10 mM Tris–HCl pH 8.0, 5 mM MgCl2). The obtained nuclei were incubated with 3.5 μL Tn5 transposomes in 40 μL TTBL buffer (TruePrep DNA Library Prep Kit V2 for Illumina, Vazyme Biotech co., ltd, TD501) at 37 °C for 30 min without rotation. The integration products were purified using NEB MonarchTM DNA Cleanup Kit (T1030S) and then amplified for 10–13 cycles using the NEBNext Ultra II Q5 master mix.

### DAP-seq library preparation

DAP-seq was performed as described by Pei et al. (Pei et al. 2023b) with modifications. The NEB Next® DNA Library Prep Master Mix set for Illumina Kit (NEB #E6040S) was used to prepare the DAP-Seq gDNA library. The *BrKAN2.1* gene was fused to the HaloTag using the pIX-halo vector, and the fusion protein (HALO-BrKAN2.1) was expressed using TNT SP6 Coupled Reticulocyte Lysate System (Promega, USA). The fusion protein was purified with Magne-HALO Tag beads (Promega). The purified HALO-BrKAN2.1 protein and 500 ng of gDNA library were co-incubated in 50 μL PBST buffer (1 × PBS with 0.005% Nonidet P-40) for 1 h at room temperature with gentle shaking. Beads were washed eight times with PBST, followed by incubation with 1 μL Tn5 transposomes (Vazyme, TD501) in 50 μl of TTBL buffer at 55 °C for 10 min. DNA was recovered using New Miniquick Purification Kit (Zomanbio, China, ZPY201) and amplified with KAPA HiFi HotStart ReadyMix (KK2601) for 10–13 cycles. Amplified libraries were purified with VAHTS DNA Clean Beads (Vazyme, N411) to remove primers. Library concentration was adjusted based on fragment size and target read count. Mock DAP-Seq libraries (negative controls) were prepared similarly, but without protein added to the beads.

### Quantitative reverse transcription PCR (RT-qPCR) analysis

Total RNA was extracted form *B. rapa* (R-O-18) leaves treated with VIGS. Reverse transcription was performed using *Evo M-MLV* (AG11705, Accurate Biology, China), followed by qPCR using SYBR Green *Pro Taq* HS (AG11701, Accurate Biology, China). RT-qPCR was performed on an ABI QuantStudio 12 K Flex Real-Time PCR system (Applied Biosystem, Foster City, CA, USA). Relative gene expression levels were calculated using the 2^−ΔΔCt^ method, with *BrACTIN3* as an internal control.

### Auxin treatment

Chinese cabbage (Chiifu) plants were grown in a greenhouse at 25 °C under a 16 h light and 8 h dark photoperiod. When the plants reached 7 leaves, they were sprayed with 10^–3^ mM auxin (0.15% Triton X-100) once a week. Leaf phenotypes were observed after three times of auxin treatment.

### Generation and analysis of transgenic plants overexpressing *BrKAN2s*

The coding sequences of *BrKAN2s* were amplified from Chiifu-401–42 cDNA and cloned into the pCAMBIA1300 binary vector. Recombinant vectors were transformed into *Arabidopsis* Col-0 via the floral-dip method. Transgenic lines were selected on MS media with 50 mg/L hygromycin and transplanted into soil. The T_3_ generation was used for phenotypic analysis. A minimum of ten seedlings per line were investigated. The lengths of leaves and petioles were measured with a Vernier caliper. Leaf sections were prepared via paraffin embedding and observed under a microscope.

### Subcellular localization

Subcellular localization was analyzed using a transient expression system in *Nicotiana Benthamian*. *Agrobacterium tumefacient* strain GV3101 harboring 35S:GFP or 35S:*BrKAN2.1*-GFP was separately mixed with Agrobacterium carrying a nuclear-localized mCherry construct at a 1:1 ratio and infiltrated into leaves of 5-week-old plants. At 48 h after infiltration, leaf tissues were excised, with the main veins removed, and examined by confocal microscopy. Fluorescence signals were observed using a Zeiss LSM 880 confocal laser scanning microscope (Carl Zeiss, Germany).

### Yeast-based assays for transcriptional activation/inhibition

Using the ClonExpressII One-Step Cloning Kit (Vazyme, China), the CDS of *BrKAN2.1* was inserted into the pGBKT7 vector digested with B*am*HI and S*ac*I. Subsequently, pGBKT7-VP16, pGBKT7, and pGBKT7-*BrKAN2.1* were transformed into AH109 yeast competent cells (Coolaber, China) respectively. The transformed cells were spread onto SD/-Trp medium (Coolaber, China) and incubated at 29 °C for 3-5 days to observe growth. Single colonies from the SD/-Trp plates were then picked and subjected to serial dilutions (1×, 10×, 100×). These diluted samples were spotted onto SD/-Trp, SD/-Trp/-His, and SD/-Trp/-His + X-α-gal solid media (Coolaber, China) for further verification.

### Mutants screening of *BrKAN2s*

We previously established an EMS-mutagenized mutant library of Chinese cabbage and sequenced approximately 2000 individual lines. From this library, two allelic *BrKAN2.1* mutants and one *BrKAN2.3* mutant with premature stop codons were identified. PCR amplification and Sanger sequencing confirmed the presence of these mutations in the respective loci.

### Virus-induced gene silencing

*BrKAN2*-silenced plants were generated using Xiao’s method (Xiao et al. 2020). A 323 bp fragment, homologous to both *BrKAN2.1* and *BrKAN2.3* coding sequence, was inserted into PCVA vector. The PCVA-PDS construct served as a reference. The constructs, PCVA-*BrKAN2* and empty vector, were transformed into *B. rapa* (R-O-18) with two cotyledons and transplanted into a climate for 24 h in the dark. After two weeks, when the leaves of PCVA-*PDS* plants turned white, samples from PCVA-*BrKAN2* plants were collected and stored in −80 °C for RT-qPCR.

### Electrophoretic mobility shift assay (EMSA)

The *BrKAN2.1* was inserted into the vector pCold-Halo. The recombinant protein was expressed in Promega SP6 High-Yield Protein Expression System (L3261). Probe fragments from the promoter of *BrARF5* and *BrSAW2* were synthesized by Shanghai Sangon Co., Ltd. The EMSA chemiluminescence kit was purchased from Coolaber Technology (Beijing, China, MH104). The binding reactions containing BrKAN2.1 protein with *BrARF5* or *BrSAW2* probes were performed in 10 × Binding Buffer at 25 °C for 20 min, following the manufacturer's protocol. Protein-DNA complexes were detected using the Odyssey dual-color infrared laser imaging system.

### ATAC-seq data processing

The raw Illumina high-throughput sequencing data (FASTQ file) were initially assessed for quality using FastQC. Adapter sequences and poor-quality reads were removed using Trimmomatic v0.38. The remaining reads were mapped to the *B. rapa* reference genome (v3.0) using Bowtie2 v2.4.5. Removal of non-nuclear comparisons from SAM files using SAMTools v1.14, and the resulting BAM files were sorted and PCR duplicates removed with the parameter “-bS -q 20”. Deeptools was used to obtain the reads distribution in whole genome and plot the distribution heatmap, Spearman’s correlation and PCA. Peaks calling was performed using MACS2 v2.2.6 with an initial threshold of “-B -q 0.001 -fe 3 -shift” to identify open chromatin regions. We reduced background noise from technique by adjusting the "-fe" parameters. We use AIAP v1.1 to assess the quality of peaks (Liu et al. 2021). DiffBind v3.8.4 was used to identify the differential accessible chromosome regions. The GO database “THE GENE ONTOLOGY RESOURCE (https://geneontology.org/)” was utilized. The findMotifsGenome.pl subcommand from Homer (v4.11.1) was utilized to identify motifs in ATAC-seq data across three developmental stages with the parameters “-size 200 -len 8 -mset plants”.

### DAP-seq data processing

Raw reads were assessed with FastQC v0.12.1 and trimmed by Trimmomatic v0.38 with default parameter. Trimmed reads were aligned to *B. rapa* reference genome (v3.0) using Bowtie2 v2.4.5 with the parameters: ‘‘bowtie2 -X 1000 -p -5 -S’’. Aligned reads were sorted and PCR duplicates were removed using SAMtools v1.14. Peaks were called with MACS2 v2.2.6 using the parameters: “macs2 callpeak -keep-dup all -g”. Transcription factor binding motifs were predicted using Homer. Bigwig files were used to visualize peaks in the IGV. The findMotifsGenome.pl subcommand from Homer (v4.11.1) was utilized to identify motifs in DAP-seq data with the parameters “-size 200 -len 8 -mset plants”. Additionally, MEME-ChIP was employed to assist in motif identification and generate visualization logos.

## Supplementary Information

Additional file 1: Fig. S1 ATAC-seq data quality control. Fig. S2 Functional enrichment analysis of genes associated with differential ACRs between the seedling and rosette stages and between the rosette and heading stages. Fig. S3 Number of TFs identified from differential ACRs. Fig. S4 Expression heatmaps of leaf polarity related genes across different stages in Chinese cabbage. Fig. S5 The genotype of the structural variation in the *BrKAN2.1* gene in 524 accessions including 350 heading and 184 non-heading *B. rapa*. Fig. S6 The genotype of the structural variation in the *BrKAN2.3* gene in 524 *B. rapa* accessions including 350 heading and 184 non-heading *B. rapa*. Fig. S7 Sequence alignment and domain analysis of KAN2 protein in *Arabidopsis thaliana* and *B. rapa*. Fig. S8 Evolutionary analysis of the KANADI family in *Arabidopsis* and *Brassica rapa*, and *Brassica olereacea*. Fig. S9 BrKAN2 regulates leaf morphology in both Chinese cabbage and *Arabidopsis*. Fig. S10 Quality control of DAP-seq data. Fig. S11. Relative expression levels of *BrKAN2.1* target genes in PCVA-*BrKAN2* and PCVA plants.

Additional file 2: Table S1. ATAC-seq data volume statistics in three stages. Table S2. Peak calling QC in three stages. Table S3. TFs associated with leaf polarity in high accessibility group at rosette vs seedling stage. Table S4. TFs associated with leaf polarity in low accessibility group at heading vs rosette stage. Table S5. Motifs enriched in differential accessibility regions at rosette vs seedling stage. Table S6. Motifs enriched in differential accessibility regions at heading vs rosette stage. Table S7. Significant mutations in the *BrKAN2* gene body and promoter. Table S8. Similarities (%) between *KAN2* genes in *Arabidopsis thaliana* and *Brassica rapa*. Table S9. Comparison of physiological indices between wild-type and mutant. Table S10. Target gene of ethylene response pathway. Table S11. Target gene of plant organ development pathway. Table S12. Target gene of auxin response pathway. Table S13. Important cis-elements on *KAN2* genes promoter in *Arabidopsis thaliana* and *Brassica rapa*.

## Data Availability

All sequencing data generated for this study have been submitted to the NGDC (https://ngdc.cncb.ac.cn/) Sequence Read Archive under accession number PRJCA033732.

## Abbreviations

KAN: KANADI
DAP-seq: DNA Affinity Purification Sequencing
ATAC-seq: Assay for Transposase-Accessible Chromatin with high throughput sequencing
RNA-seq: RNA-sequencing
ChIP-seq: Chromatin Immunoprecipitation followed by Sequencing
ACRs: Accessible chromatin regions
dACRs: Differential accessible chromatin regions
CREs: Cis-regulatory Elements
VIGS: Virus-Induced Gene Silencing
EMSA: Electrophoretic Mobility Shift Assay
SAM: Shoot Apical Meristem
PCA: Principal Component Analysis
TSS: Transcription Start Site
TTS: Transcription Termination Site
GO: Gene Ontology
TF: Transcription Factor
IGV: Integrative Genomics Viewer
ARF: Auxin Response Factor
ERF: Ethylene Response Factor
IAA: Indole-3-acetic Acid
SAW: SAWTOOTH
REV: REVOLUTA
GA: Gibberellic Acid
JA: Jasmonic Acid
RT-qPCR: Quantitative Reverse Transcription PCR
