# Supplementary material for: Dynamic chromatin accessibility reveals BrKAN2 as a key regulator of Chinese cabbage leaf heading
Source: Mol Hortic. 2026 Jul 8;6:58. doi: 10.1186/s43897-026-00239-6 (PMC13343676; doi:10.1186/s43897-026-00239-6)
Supplement: Supplementary file 1 — Additional file 1: Fig. S1 ATAC-seq data quality control. Fig. S2 Functional enrichment analysis of genes associated with differential ACRs between the seedling and rosette stages and between the rosette and heading stages. Fig. S3 Number of TFs identified from differential ACRs. Fig. S4 Expression heatmaps of leaf polarity related genes across different stages in Chinese cabbage. Fig. S5 The genotype of the structural variation in the BrKAN2.1 gene in 524 accessions including 350 heading and 184 non-heading B. rapa. Fig. S6 The genotype of the structural variation in the BrKAN2.3 gene in 524 B. rapa accessions including 350 heading and 184 non-heading B. rapa. Fig. S7 Sequence alignment and domain analysis of KAN2 protein in Arabidopsis thaliana and B. rapa. Fig. S8 Evolutionary analysis of the KANADI family in Arabidopsis and Brassica rapa, and Brassica olereacea. Fig. S9 BrKAN2 regulates leaf morphology in both Chinese cabbage and Arabidopsis. Fig. S10 Quality control of DAP-seq data. Fig. S11. Relative expression levels of BrKAN2.1 target genes in PCVA-BrKAN2 and PCVA plants. [file 43897_2026_239_MOESM1_ESM.docx]

**Supplementary Figures**


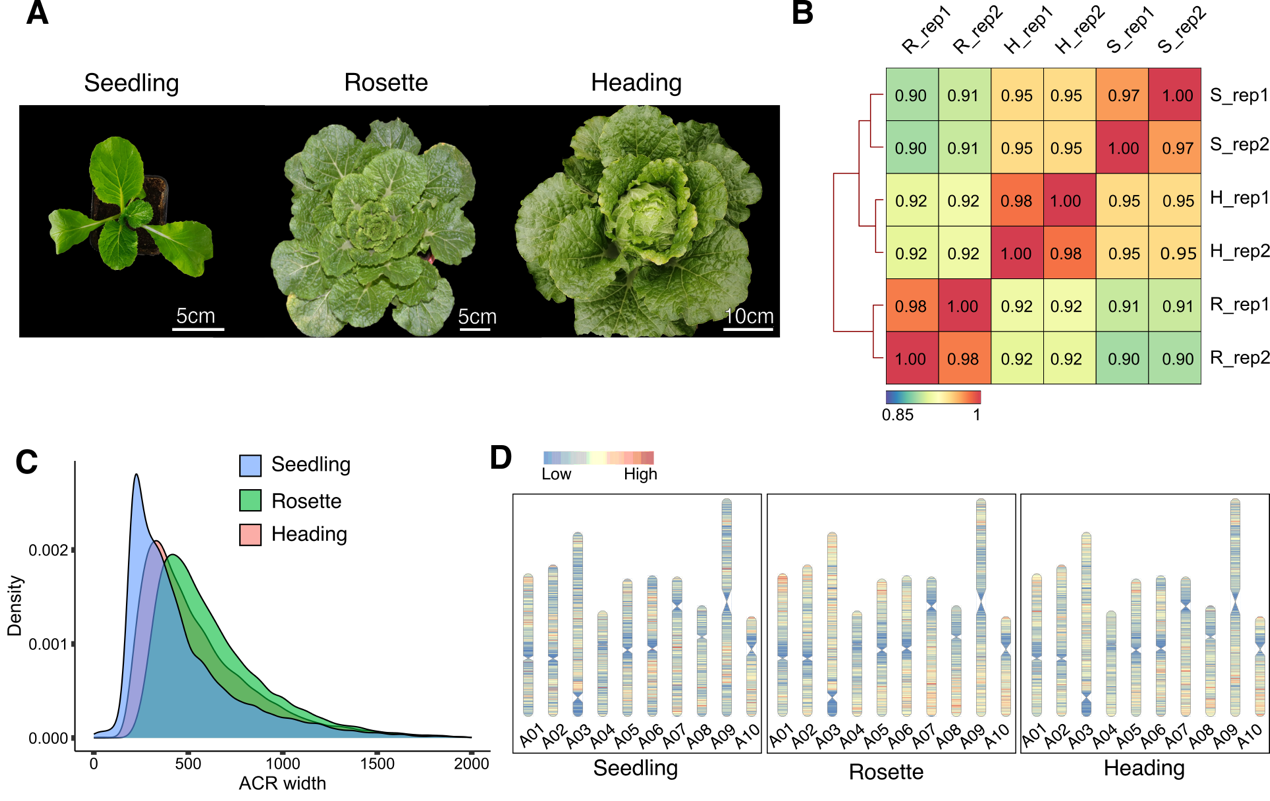


**Fig. S1** ATAC-seq data quality control. **(A)** Representative phenotype of Chinese cabbage at the seedling, rosette, and heading stages. **(B)** The heatmap plot shows the spearman correlation coefficient between biological replicate samples at the seedling (S), rosette (R), and heading (H) stages. The reads number per 10 kb was used for read intensity evaluation. **(C)** Distribution of ACR width at three stages. **(D)** Distribution of ACRs at three stages throughout the *B. rapa* genome.


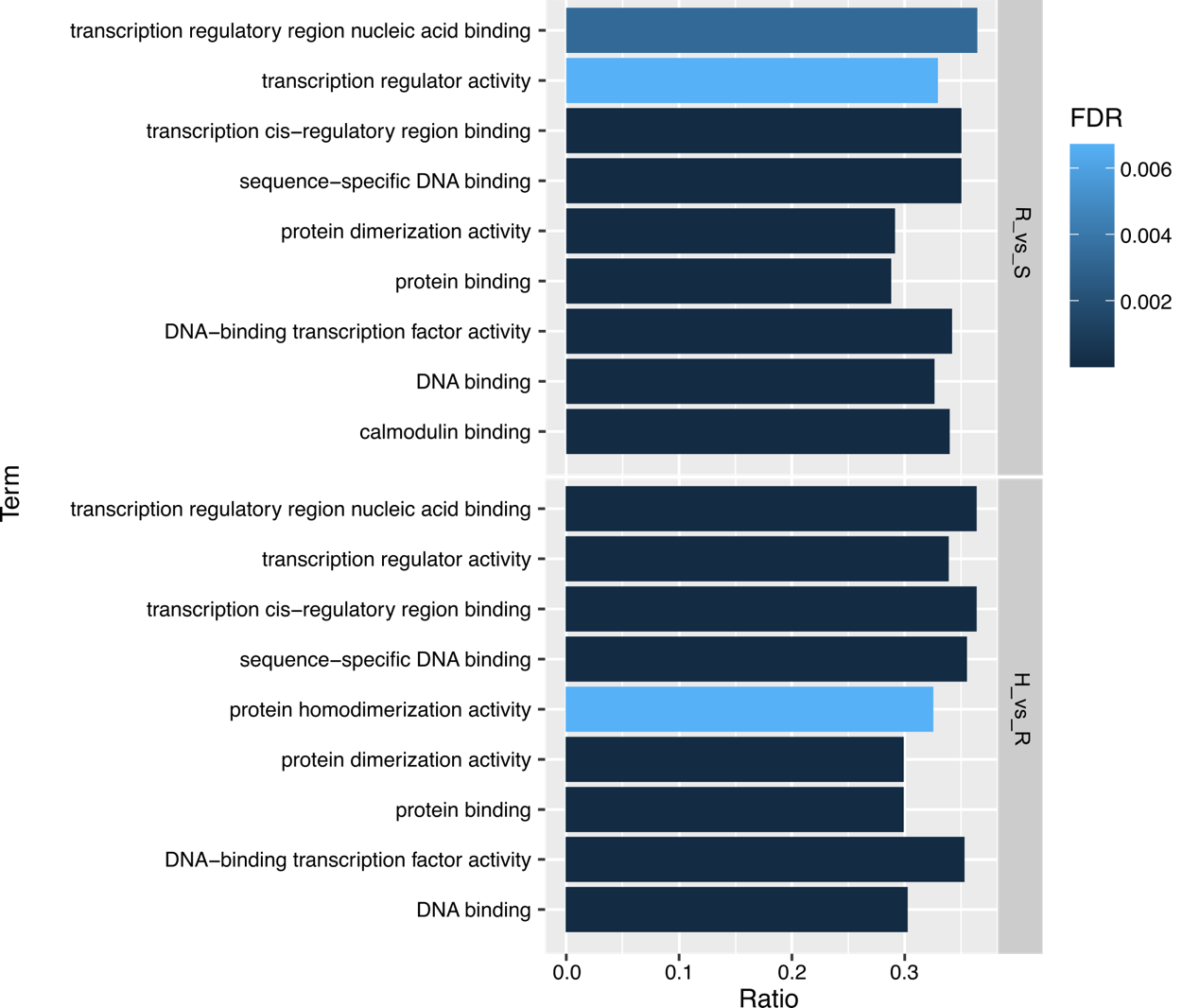


**Fig. S2** Functional enrichment analysis of genes associated with differential ACRs between the seedling and rosette stages and between the rosette and heading stages.


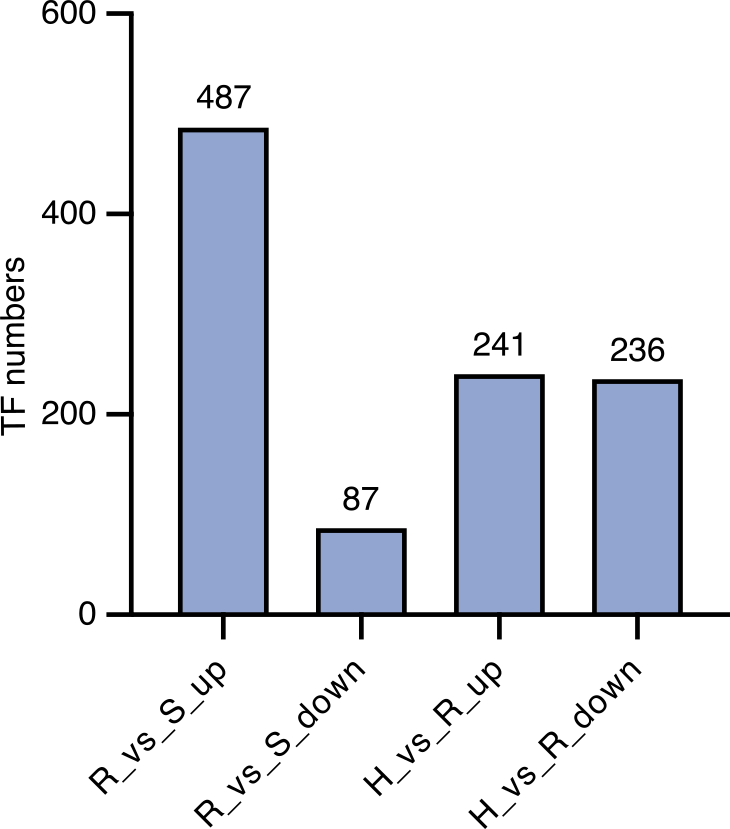


**Fig. S3** Number of TFs identified from different ACRs.


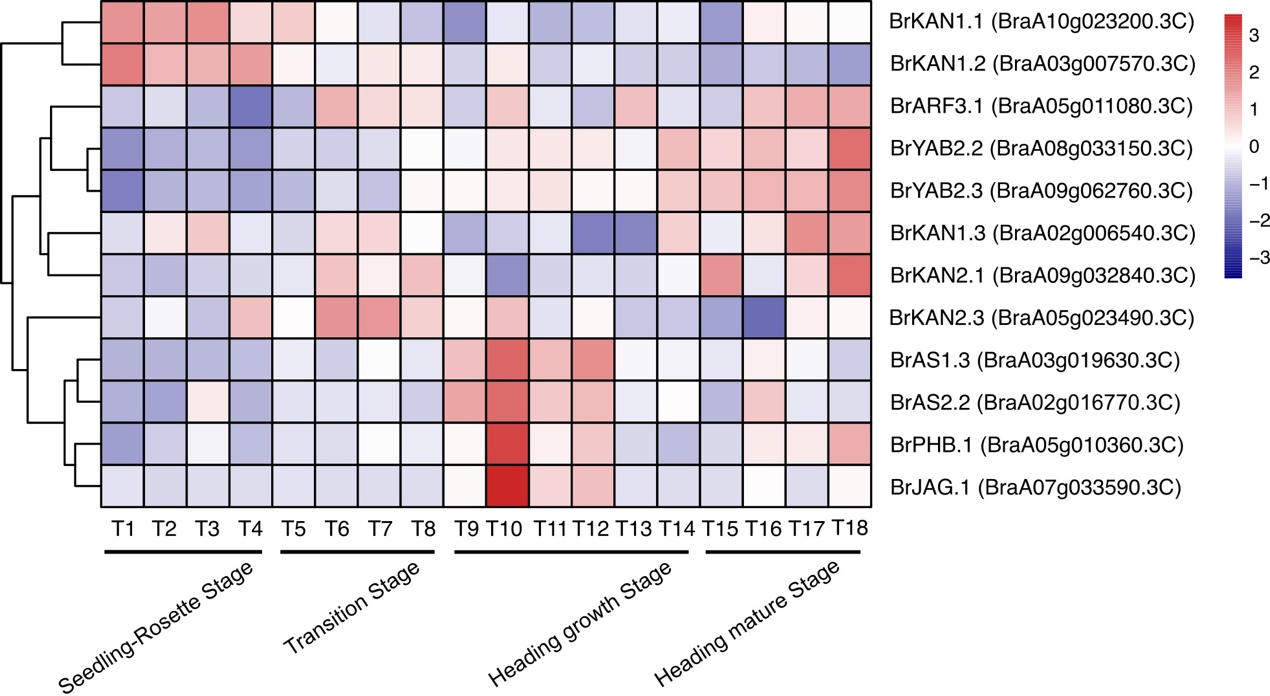
**Fig. S4** Expression heatmaps of leaf polarity related genes across differential stages in Chinese cabbage.


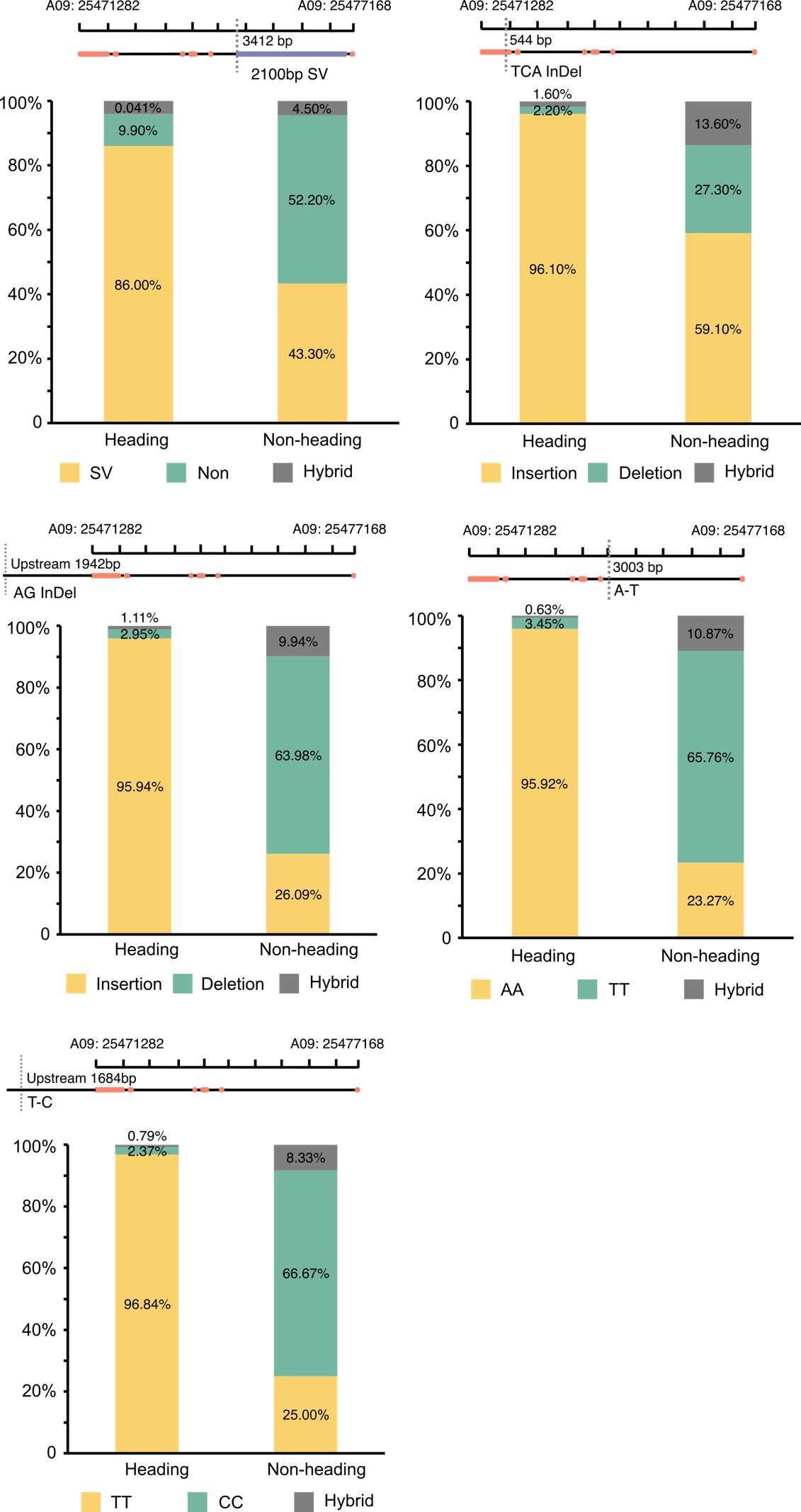


**Fig. S5** The genotype of the structural variation in the *BrKAN2.1* gene in 524 accessions including 350 heading and 184 non-heading *B. rapa*. These five bar plots represent the proportions of SV insertions, TCA insertion or deletion, AG insertion or deletion, A/T substitutions and T/C substitutions in heading and non-heading *B. rapa*, respectively.


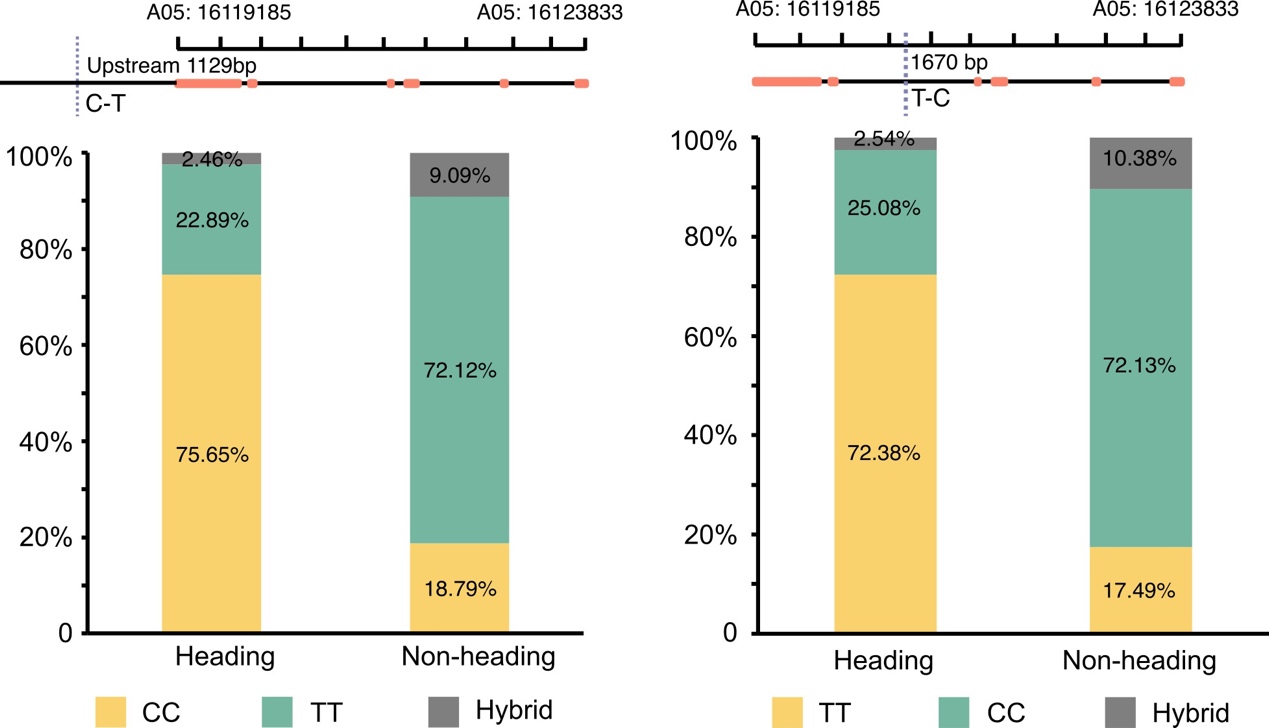
**Fig. S6** The genotype of the structural variation in the *BrKAN2.3* gene in 524 *B.rapa* accessions including 350 heading and 184 non-heading *B. rapa*. These two bar plots represent the proportions of C/T substitutions and T/C substitutions in heading and non-heading *B. rapa*, respectively.


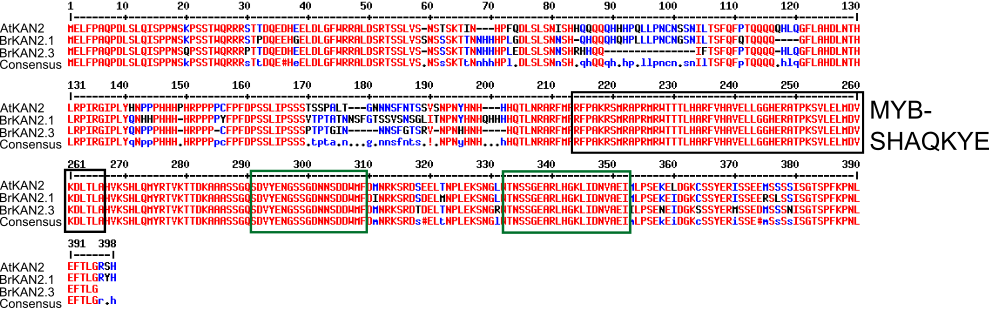


**Fig. S7** Sequence alignment and domain analysis of KAN2 protein in *Arabidopsis thaliana* and *B. rapa*.


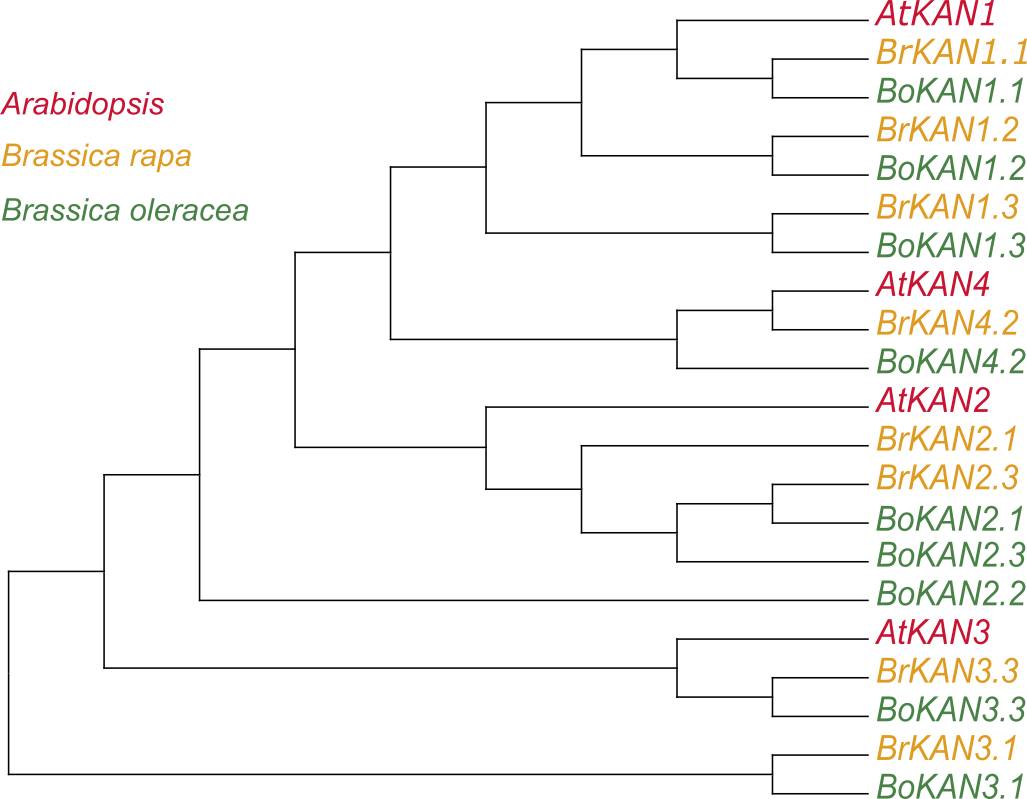
**Fig. S8** Evolutionary analysis of the KANADI family in *Arabidopsis* and *Brassica rapa*, and *Brassica olereacea*. The colors indicate the source species: red represents KANADI family genes in *Arabidopsis*, yellow represents those in *Brassica rapa*, and green represents those in *Brassica oleracea*. Branch lengths correspond to the evolutionary distance between KANADI family genes. The tree was constructed using the Neighbor-Joining method in MEGA-X, with bootstrap support based on 1000 replicates.


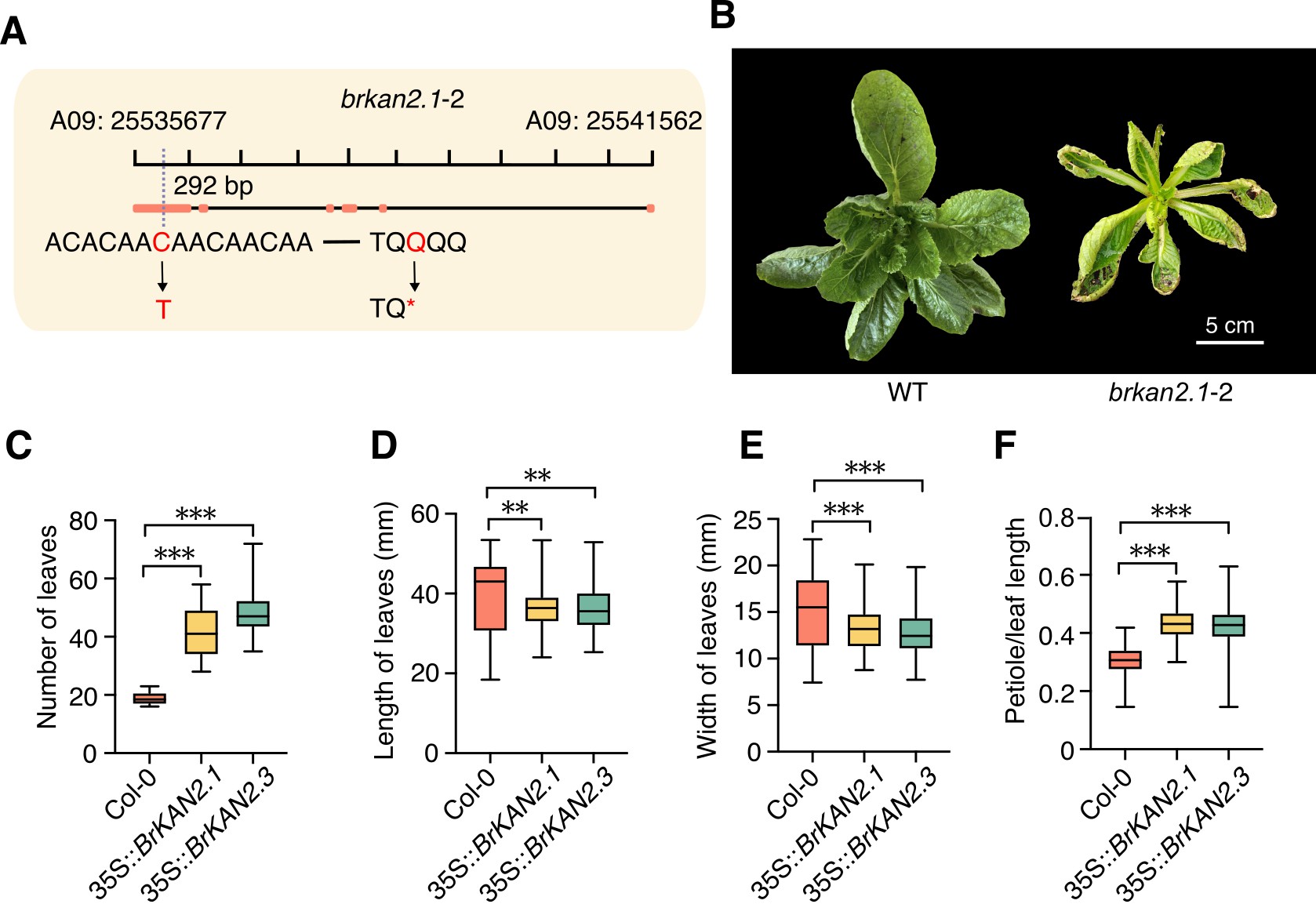


**Fig. S9** *BrKAN2* regulates leaf morphology in both Chinese cabbage and Arabidopsis. **(A)** Schematic diagram of mutation sites in *BrKAN2.1* genes. **(B)** Representative plant images of wild-type and *brkan2.1*. **(C-F)** Comparsion of leaf numbers, leaf length, left width, and petiole-to-leaf length ratio between 35S::*BrKAN2* and Col-0 (*P ≤ 0.05; **P ≤ 0.01; and ***P ≤ 0.001).


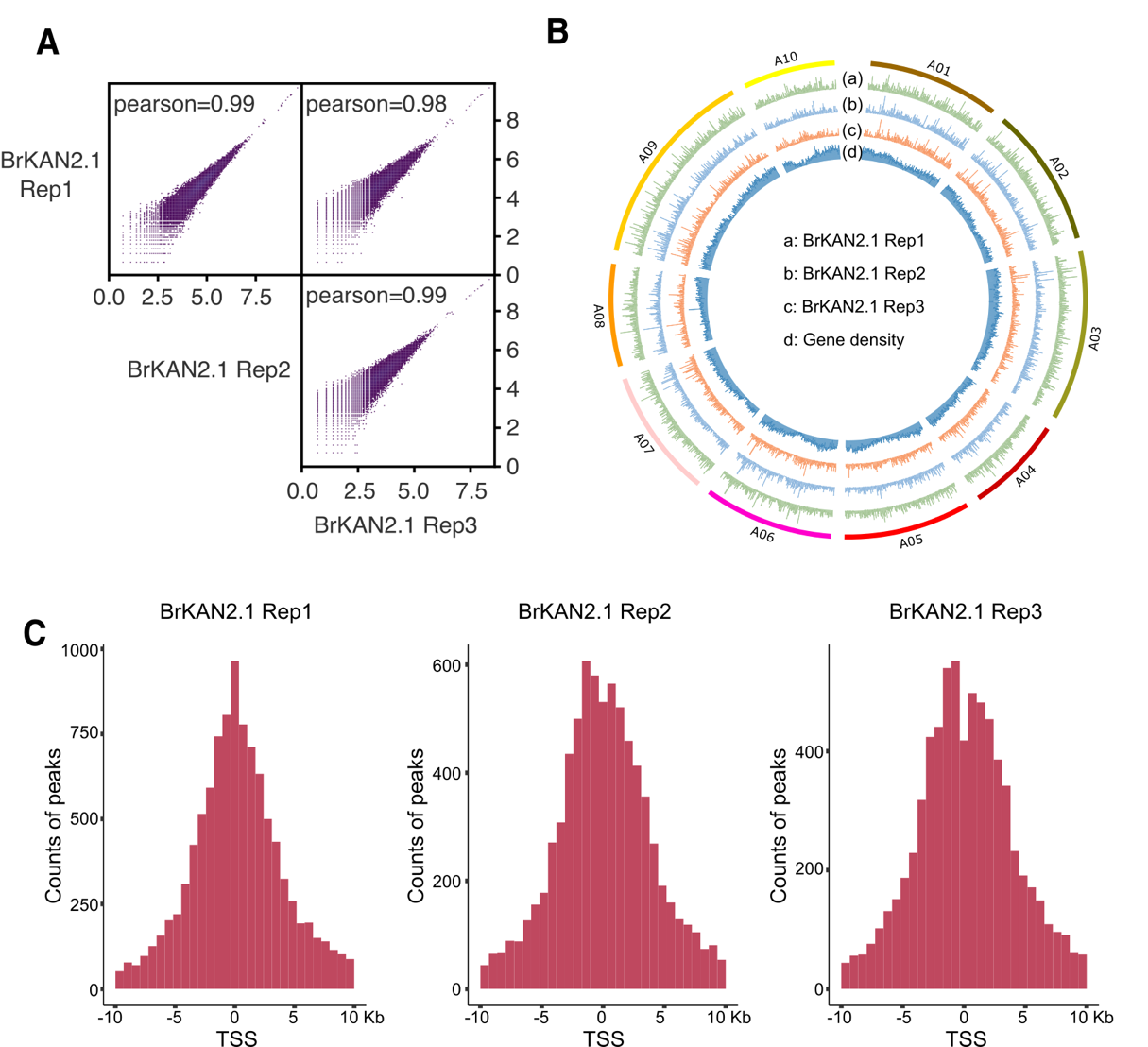


**Fig. S10** Quality control of DAP-seq data. **(A)** Pearson correlation coefficient analysis of reads intensity between replicates for DAP-seq. The reads number per 10kb was used for reads intensity evaluation. (**B**) BrKAN2.1 binding signals across the genome from three biological replicates. **(C)** Distance from the center of the binding site to TSS in three biological replicates.


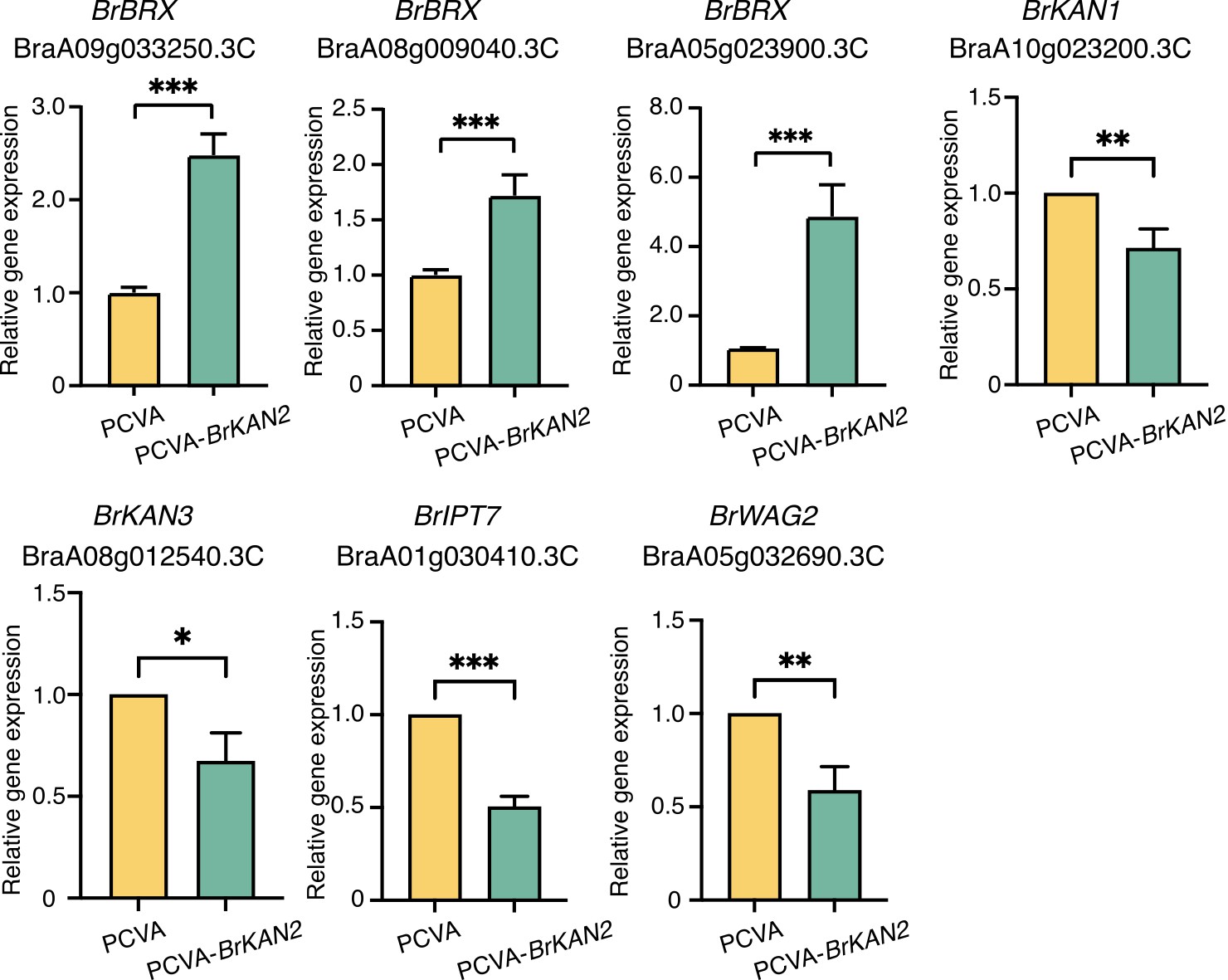
**Fig. S11.** Relative expression levels of BrKAN2.1 target genes in PCVA-*BrKAN2* and PCVA plants. The error bars represent the standard deviations of three independent biological repeats. The asterisks represent significant differences via T-tests (*p ≤ 0.05; **p ≤ 0.01; and ***p ≤ 0.001).
